# Phenotypic Expression and Stability in a Large-Scale Field Study of Genetically Engineered Poplars Containing Sexual Containment Transgenes

**DOI:** 10.3389/fbioe.2018.00100

**Published:** 2018-08-03

**Authors:** Amy L. Klocko, Haiwei Lu, Anna Magnuson, Amy M. Brunner, Cathleen Ma, Steven H. Strauss

**Affiliations:** Department of Forest Ecosystems and Society, Oregon State University, Corvallis, OR, United States

**Keywords:** RNAi, *Populus*, dominant negative mutations, gene flow, biosafety

## Abstract

Genetic engineering (GE) has the potential to help meet demand for forest products and ecological services. However, high research and development costs, market restrictions, and regulatory obstacles to performing field tests have severely limited the extent and duration of field research. There is a notable paucity of field studies of flowering GE trees due to the time frame required and regulatory constraints. Here we summarize our findings from field testing over 3,300 GE poplar trees and 948 transformation events in a single, 3.6 hectare field trial for seven growing seasons; this trial appears to be the largest field-based scientific study of GE forest trees in the world. The goal was to assess a diversity of approaches for obtaining bisexual sterility by modifying RNA expression or protein function of floral regulatory genes, including *LEAFY, AGAMOUS, APETALA1, SHORT VEGETATIVE PHASE*, and *FLOWERING LOCUS T*. Two female and one male clone were transformed with up to 23 different genetic constructs designed to obtain sterile flowers or delay onset of flowering. To prevent gene flow by pollen and facilitate regulatory approval, the test genotypes chosen were incompatible with native poplars in the area. We monitored tree survival, growth, floral onset, floral abundance, pollen production, seed formation and seed viability. Tree survival was above 95%, and variation in site conditions generally had a larger impact on vegetative performance and onset of flowering than did genetic constructs. Floral traits, when modified, were stable over three to five flowering seasons, and we successfully identified RNAi or overexpression constructs that either postponed floral onset or led to sterile flowers. There was an absence of detectable somaclonal variation; no trees were identified that showed vegetative or floral modifications that did not appear to be related to the transgene added. Surveys for seedling and sucker establishment both within and around the plantation identified small numbers of vegetative shoots (root sprouts) but no seedlings, indicative of a lack of establishment of trees via seeds in the area. Overall, this long term study showed that GE containment traits can be obtained which are effective, stable, and not associated with vegetative abnormalities or somaclonal variation.

## Introduction

Trees provide humans with a variety of useful products, including wood, fiber, energy, and food. In addition to these tangible products, trees also provide ecological services, such as carbon capture, water purification, and by serving as keystone species that promote biodiversity. Plantation ecosystems, though generally less diverse than wild ones, can also promote biodiversity and help to reduce pressure on native forests (Barlow et al., 2007; Brockerhoff et al., 2008).

Genetic improvement is extremely important to orchard and plantation management. Although a wide variety of biotechnologies are used for tree breeding, genetic engineering is of great interest because it bypasses the long generation cycle and intolerance to inbreeding of trees, and allows traits to be added or modified without significant background changes to commercially valuable clones. Examples of genetically engineered (GE) trees include agricultural species such as *Carica papaya* (papaya) (Fitch et al., 1993) and *Malus domestica* (apple) (Boresjza-Wysocka et al., 1999; Murata et al., 2000), forestry species including *Populus* (poplars) (Meilan et al., 2002; Klocko et al., 2014; Yang et al., 2015; Ault et al., 2016), *Eucalyptus* species (eucalypts) (Harcourt et al., 2000; Matsunaga et al., 2012), and even wild and ornamental trees such as *Castanea dentata* (American chestnut) and *Ulmus americana* (American elm) (Newhouse et al., 2007; Sherif et al., 2016) (Maynard et al., 2009; Zhang et al., 2013). However, most of these varieties, exceptions being deregulated virus resistant papaya and non-browning Arctic apple, are not grown commercially (Strating, 1996; Waltz, 2015). This limited uptake by growers and consumers is not due to a lack of success of the traits of interest, but rather due to the controversy surrounding the GE process used to produce them.

A major concern for GE trees is gene flow; the spread of trees or their gametes beyond the boundaries of plantings. Similar concerns about gene flow apply for exotic tree species, which have become invasive in a number of instances (Richardson and Rejmanek, 2011) and could thus benefit from the same containment technologies as discussed for GE trees. Unlike many crops, most trees are perennial, long-lived, and weakly-domesticated—exacerbating gene flow concerns. Gene flow can occur through localized vegetative spread in some species, such as by shoots from spreading roots, and by rooting of detached branches, such as in various species of poplars. In most tree species, however, long-distance spread occurs mostly via sexual reproduction through the movement of pollen or seeds.

Studies of GE tree species have shown that gene flow can and does occur, and its extent varies widely among species and environments. For example, poplar is a wind-pollinated, outcrossing species with potential for long distance spread by pollen and its cottony seeds. Models for predicted gene flow in poplar show that fertility is a key factor for influencing spread, as is the fitness effect of the trait encoded by a transgene (DiFazio et al., 2012). A recent study of insect resistant cry1Ac poplar in China quantified the amount of gene flow between male cry1Ac trees and female trees in the surrounding plantations. They found that the rate of GE seed formation varied from 0.00 to 0.16% of seeds, and no GE seeds were found at distances greater than 500 m from the male trees (Hu et al., 2017). In addition, they also found that seeds purposefully planted in the field failed to germinate unless they received purposeful intervention, such as irrigation, indicating a low risk of seedling establishment. Other studies of transgene flow are from fruit tree species. GE plum pox resistant trees have been developed and are deregulated, but are not in commercial production (Ravelonandro et al., 1997; USDA, 2015). Plum flowers have bee-mediated pollen transfer, and a low rate of gene flow from GE trees (up to 0.215–0.117% of tested embryos), which drops off with distance (Scorza et al., 2013). Even fruit trees that are obligate outcrossers, such as apple, have distance-limited movement of pollen by bees. One study found that at distances of greater than 146 meters, no GE seeds were detected (Tyson et al., 2011). One of the few commercialized GE trees is papaya (Gonsalves, 2006). Field evaluation of pollen flow between GE and conventional stands showed a very low rate of pollen transfer, between 0.3 and 1.3% of embryos tested (Gonsalves et al., 2012). While papaya is wind-pollinated, the varieties grown were bisexual, and were likely self-pollinating. For the fruit trees species detailed above, only pollen-mediated transgene dispersal was studied. The fruits produced by these species are large and fleshy, and may or may not undergo long-distance dispersal in field conditions, depending on the species and nature of foraging by animal dispersers (e.g., birds vs. mammals).

There is a paucity of field data for GE forest trees, and much of it comes from short term trials (reviewed in Strauss et al., 2017). Desired data include assessment of measured ecological impacts of GE trees as compared to non-GE tress, GE tree performance such as growth and survival, and the effects of the specific engineered traits on commercial properties. While laboratory and greenhouse trials are useful for initial assessments, it is known that these results rarely match those obtained in the field. For example, a field and greenhouse test of reduced lignin GE poplar trees found that tree form, size, and wood characteristics differed dramatically between greenhouse and field conditions (Voelker et al., 2011). Similar results have been reported in other studies (e.g., Viswanath et al., 2012). Unfortunately, permits for field trials are often difficult to obtain, in part due to the risk of gene flow into feral and wild populations. Unless flowering is explicitly allowed by permits, trees must be terminated before reaching maturity. However, juvenile trees are known to differ in trait expression, such as for wood characteristics from adult trees (Zobel and Sprague, 1998). Thus, in addition to enabling commercial use, a containment system could have large benefits for enabling field research.

There are several possible means to limit gene flow from trees. Non-GE methods include harvesting prior to maturity, growing varieties that cannot interbreed with nearby populations, creating wide hybrids which are sterile or have limited fertility, seeking and growing rare non-flowering individuals, and using random mutagenesis followed by screening to obtain sterile individuals (Ranney, 2004). Alternatively, genetic engineering can be used to specifically target one or more genes with predicted roles in flowering and/or floral fertility (Vining et al., 2012). Tree sterility could serve as an enabling technology for research and commercial use of trees modified for high-value traits.

This manuscript summarizes the findings from a large-scale field test of GE poplars that were modified with the goal of genetic containment. We report that several methods for direct modification of floral gene expression provide powerful and reliable means for impairing fertility, and thus for preventing or mitigating gene flow.

## Results

### Regulation and site management

**Figure 1:**
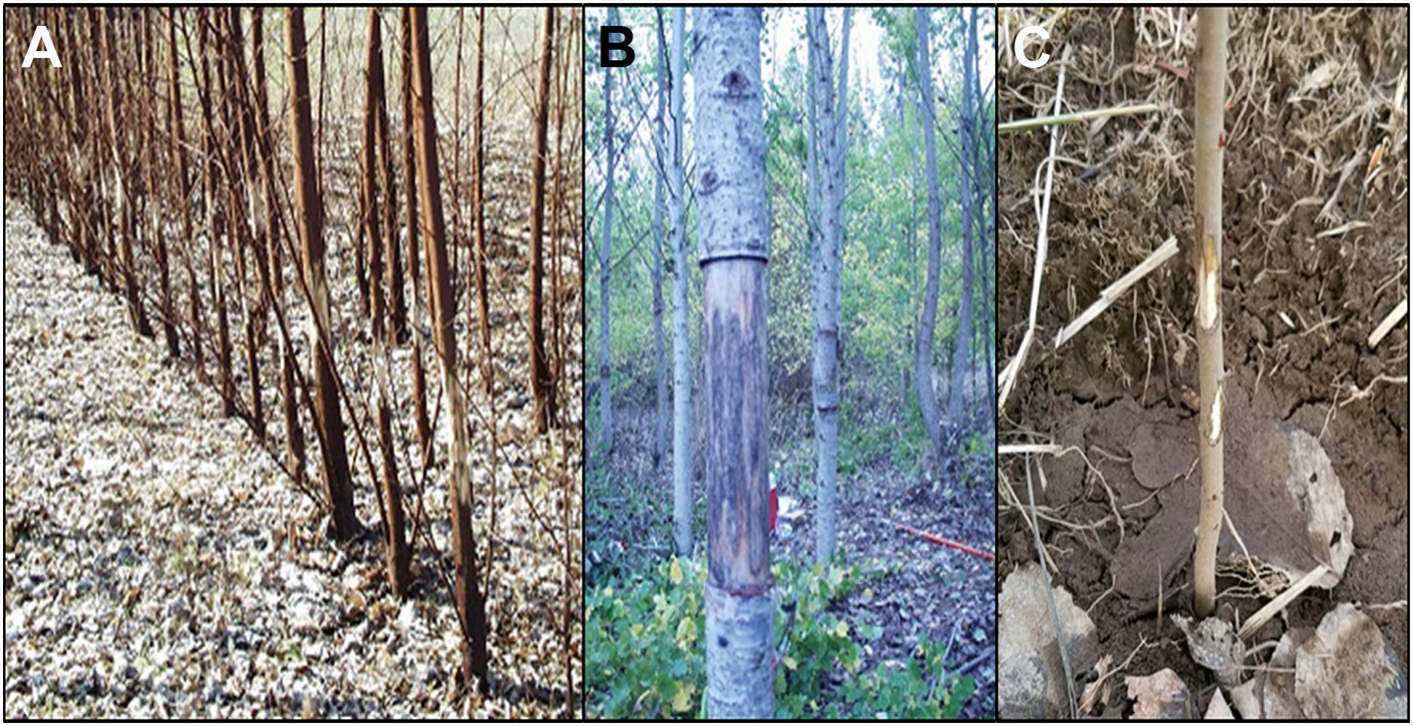
Plantation damage by human vandals and other animals can be problematic. Plantations of **(A)** young and **(B)** mature poplar trees were vandalized by humans “eco”-vandals peeling off bark in 2001. **(C)** In 2017 rodents chewed bark off of young trees.

The field trial was established in the summer of 2011 as a test of genetic constructs designed to delay or modify poplar flowering for genetic containment. In addition to genetic insights about construct effects, the experience of growing and obtaining regulatory approval for this flowering trial may be of broader interest for biosafety and field studies of GE trees. Regulatory compliance required a large amount of work before the science could even begin. All field tests of GE plants in the US require a permit from USDA APHIS prior to establishment of the plants in field. The work and costs associated with obtaining and meeting the conditions of such permits are significant barriers to field testing. In addition to costs associated with the actual scientific study of the trees, we have paid from our research budgets most of the costs of site preparation, fence maintenance, tree removal, and site monitoring after trial termination. In addition, because flowering and sexual reproduction were key traits under study, the permit had several additional monitoring requirements. The entire site was enclosed in fencing (higher than 3 m) to exclude large herbivores, mainly *Odocoileus virginianus* (whitetail deer). This fence and the gates also served as a deterrent to unauthorized humans, as did the somewhat remote site location (in an agricultural area about one mile from a town). Vandalism by humans at various GE tree locations (lab or field) is a known risk, and did occur at this and one other Oregon State University (OSU) field site in 2001 (Figure 1; Kaiser, 2001). Thankfully, human vandalism at this current site did not occur during the duration of this study. Trees that were vandalized by attempted girdling in previous trials were either removed as the trial was scheduled to be terminated (Figure 1A), or continued to grow as poplar has the ability to regrow even with removal of bark (Figure 1B). A more common source of damage is from herbivores, such as small rodents (Figure 1C), and they require constant monitoring and often trapping or toxic methods to manage them when populations are high. Trees can recover from small amounts of herbivore damage; more extensive herbivory can lead to the need for tree replacement. In one case an entire planting was destroyed during its first growing season due to an outbreak of voles at a field site; it was replanted the following year when vole populations crashed (Elias et al., 2012) Other management challenges undertaken by our research team included set up of irrigation, irrigation management and monitoring, irrigation pump repair and maintenance, and repeated weed control during the growing season.

In addition to routine management, regulatory requirements stipulate the need for frequent, documented monitoring of the site for vegetative sprouts and unanticipated tree phenotypes (the latter requires a rapid report to USDA). While this trial did not yield any unexpected traits, other trials in the same tract of land have given rise to unexpected traits. For example, a previous field trial testing GE hybrid poplar with modified gibberellic acid signaling (leading to semi-dwarfism) flowered in summer rather than in February, which is very atypical for poplar. A report of this to USDA led to immediate removal of all flowers, though the risk of pollination at that time of year was nil (Strauss et al., 2016). Other unexpected outcomes from previous trials were rare somaclonal variants (Ault et al., 2016; Strauss et al., 2016). No such variants were observed in the current trial. In addition, unanticipated environmental occurrences at the field site must be reported to USDA; in more than one instance a portion of the field site was flooded during heavy winter rains; however, no trees were lost, nor were any flowering at the times.

## Scientific goals and methods

**Figure 2:**
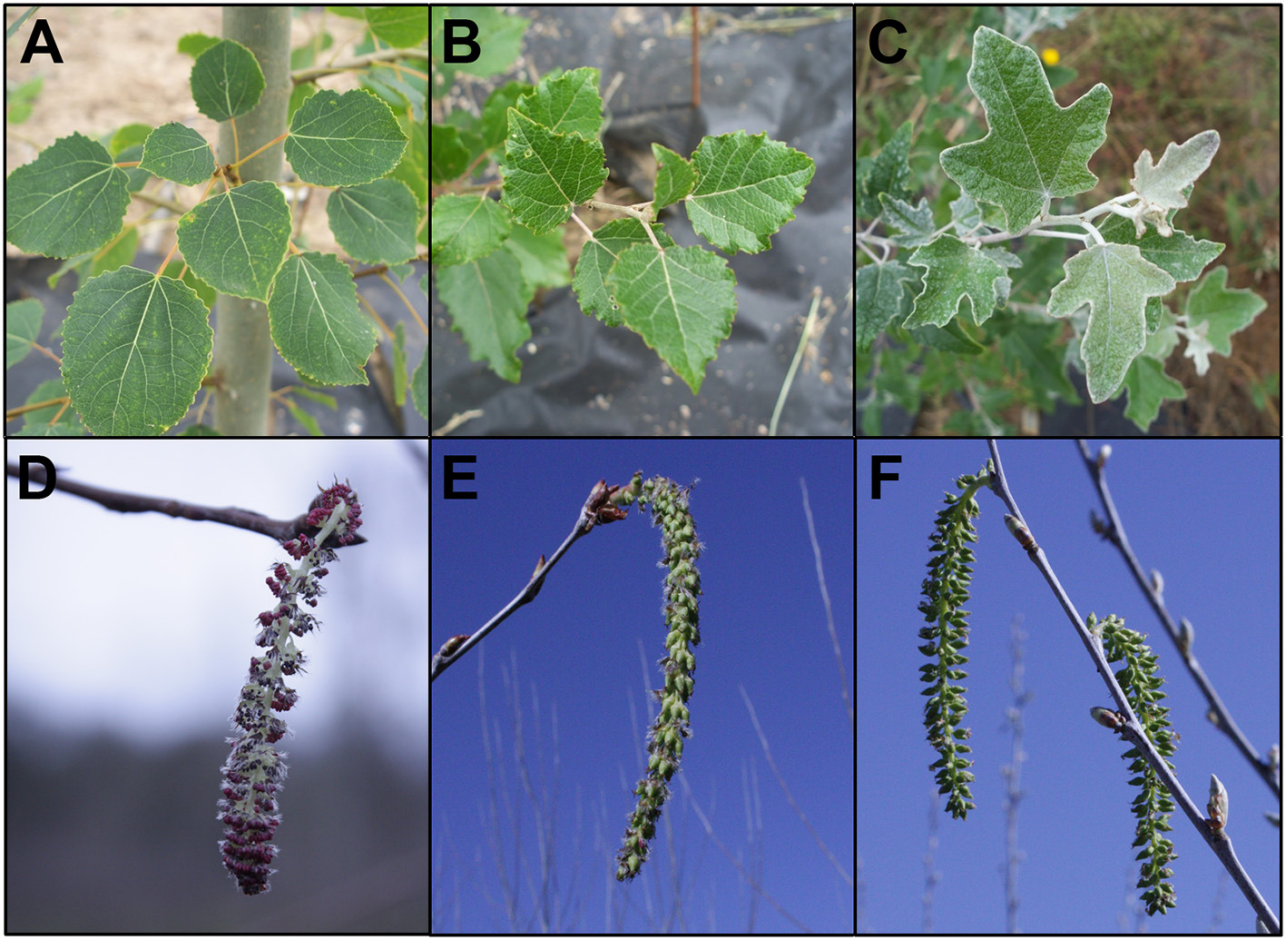
Features of the three clones of hybrid poplar studied. Male clone 353 *Populus tremula x tremuloides* had **(A)** heart-shaped leaves with fine serrations, and **(D)** staminate flowers with red anthers. Female clone 717 *Populus tremula x alba* had **(B)** triangular leaves with larger serrations and **(E)** pistilate flowers. Female clone 6K10 *Populus alba* had **(C)** lobed leaves with a tomentous abaxial surface giving a silvery appearance, and **(F)** pistilate flowers. Foliage images are from July 17, 2012, 1 year after field establishment. Flowers are from the first year of floral opening in the field. Male clone 353 flowers are from February 25, 2015, female clone 717 and female clone 6K10 flowers are from March 21, 2014.

While male sterility may be sufficient for containment of some species of plants, many trees (including poplars) have wind-dispersed seeds that can move long distances. Therefore, efficient genetic containment would require a method and gene targets that lead to bisexual sterility. Though most individual trees are unisexual, it is not uncommon to find mixed gender flowers on single trees, even if individual trees are unisexual. Because poplar is predominantly dioecious, we used male and female clones to test effects in both genders. We also used a female clone that flowers early, to speed the ability to obtain results (Figure 2). Male clone 353-53 was a hybrid, *Populus tremula x tremuloides*, and had round leaves and staminate flowers with prominent red anthers. Female clone 717-1B4 was a hybrid, *Populus tremula x alba*, and had blade shaped leaves with small serrations and pistilate flowers. Both of these clones were created by scientists at INRA in France. Female clone 6K10 was *Populus alba*, with silvery leaves and pistilate flowers, and rapid onset of flowering; it was identified by the Italian scientist Maurizio Sabatti of Tuscia University, as reviewed in Meilan et al. (2004).

Fifteen different poplar genes were selected as targets or tools for genetic containment (Table 1). At the time of vector construction, with the exception of *LEAFY* and its poplar ortholog (Weigel and Nilsson, 1995; Rottmann et al., 2000), none of the genes had been characterized in transgenic poplar and sequence data was limited to cDNAs and the initial release of the *P. trichocarpa* genome sequence. Hence, the genes were selected primarily based on knowledge of, and homology to, genes characterized in *A. thaliana*. Given the paucity of functional data about the poplar gene homologs, we selected genes from different stages in the floral pathway—from signal integration through to determination of floral organ identity—in hope of generating diverse types of sterility, some of which at least would be robust and not impart negative effects on vegetative development. In general, if there were two putative co-orthologs of an *A. thaliana* gene (as is common in poplar; e.g., *AG, AP1, FT*), we generated RNAi constructs that were predicted to target both paralogs. Twenty three constructs were designed to target these genes, either singly or in combination (Table 2). Some constructs were designed to modify the timing of floral onset or the floral abundance, while others were designed to modify floral organ identity such that anthers or carpels would instead develop as non-reproductive floral organs (Table 2). Several constructs targeted two or more different floral development genes.

**Table 1:** Genes targeted for suppression or modified expression in transgenic poplar trees.

**Gene name(s)**	**Location(s) in floral pathway**	**Poplar gene(s) from phytozome**	**Construct type(s)**
*FPF1 (FPFL1, FPFL2)*	Input from GA pathway	Potri.006G276100, Potri.018G005200, Potri.010G024500, Potri.008G209300	RNAi
*AGL20 (SOC1)*	Signal integration	Potri.014G074200	RNAi
*FT (FT1, FT2)*	Signal integration	Potri.010G179700, Potri.008077700	RNAi
*AGL24*	Signal integration Meristem determination	Potri.002G105600	OvExp, RNAi
*LFY*	Meristem determination	Potri.015G106900	RNAi
*SVP*	Meristem determination	Potri.007G010800	OvExp
*AP1 (AP1-1, AP1-2)*	Meristem determination Floral organ determination	Potri.008G098500, Potri.010G154100	DNM, RNAi
*AP3*	Floral organ determination	Potri.005G118000	RNAi
*AG (AG-1, AG-2)*	Floral organ determination	Potri.004G064300, Potri.011G075800	DNM, RNAi

*Thirteen poplar genes were selected for suppression or modification in hybrid poplar, both singly and in combination. OvExp, over expression; DNM, dominant negative mutation; RNAi, RNA interference. Genes were selected based on sequences of the initial poplar genome, gene IDs shown are from Populus trichocarpa v3.0*.

**Table 2:** Construct names and genes targeted.

**Construct name**	**Field ID**	**Construct type**	**Predicted outcome**	**Gene(s) targeted or utilized**
Control	CTR	Control	No change	None
AGL24-OE	A0	OvExp	Early flowering	AGL24
AG-M2	AM2	DNM	Delayed flowering	AtAG
AG-M3	AM3	DNM	Delayed flowering	AtAG
AP1-M2	AP2	DNM	Delayed flowering	AtAP1
AP1-M3	AP3	DNM	Delayed flowering	AtAP1
FT	FT	RNAi	Delayed flowering	FT1, FT2
SVP-OE	PS	OvExp	Delayed flowering	SVP
AGL20	A20	RNAi	Delayed flowering	AGL20 (SOC1)
AGL24	A24	RNAi	Delayed flowering	AGL24
FT:AGL20:FPF1	FAP	RNAi	Delayed flowering	FT1, FT2, AGL20 (SOC1), FPFL1
FT:AGL20	FA20	RNAi	Delayed flowering	FT1, FT2, AGL20 (SOC1)
PFPFL1	FPI	RNAi	Delayed flowering	PFPFL1
PFPFL2	FP2	RNAi	Delayed flowering	PFPFL2
PTAG	PTG	RNAi	Sterile flowers	AG1, AG2
MpTAG	MPG	RNAi	Sterile flowers	AG1, AG2 (mar)
PTAP:PTAG	PAG	RNAi	Sterile flowers	AP1-1, AP1-2, AG1, AG2
PTAP:PTLF	PAF	RNAi	Sterile flowers	AP1-1, AP1-2, LFY
PTAP	PAP	RNAi	Sterile flowers	AP1-1, AP1-2
PTD	PTD	RNAi	Sterile flowers	AP3
PTLF:PTAG	PFG	RNAi	Sterile flowers	LFY, AG1, AG2
Triple	TRP	RNAi	Sterile flowers	LFY, AG1, AG2, AP1-1, AP1-2
PTLF	PLF	RNAi	Sterile flowers	LFY
PTLF+PTAG	PFPG	RNAi	Sterile flowers	LFY, AG1, AG2

*Each genetic construct was given a unique construct name, which appeared as a shortened version on tags in the field (field ID). There were three types of constructs, OvExp, over expression; DNM, dominant negative mutation; RNAi, RNA interference. Non-transgenic control trees were indicated by control (CTR), meaning they did not undergo transformation with a genetic construct. DNM constructs were based on modified versions of A. thaliana genes predicted to inhibit the activity of their Populus homologs. The PFG and PFPG constructs were designed to target the LFY and AG genes; for PFG both gene fragments were part of a single hairpin, as were all other multi-gene targeting constructs. For PFPG two hairpins were present*.

**Figure 3:**
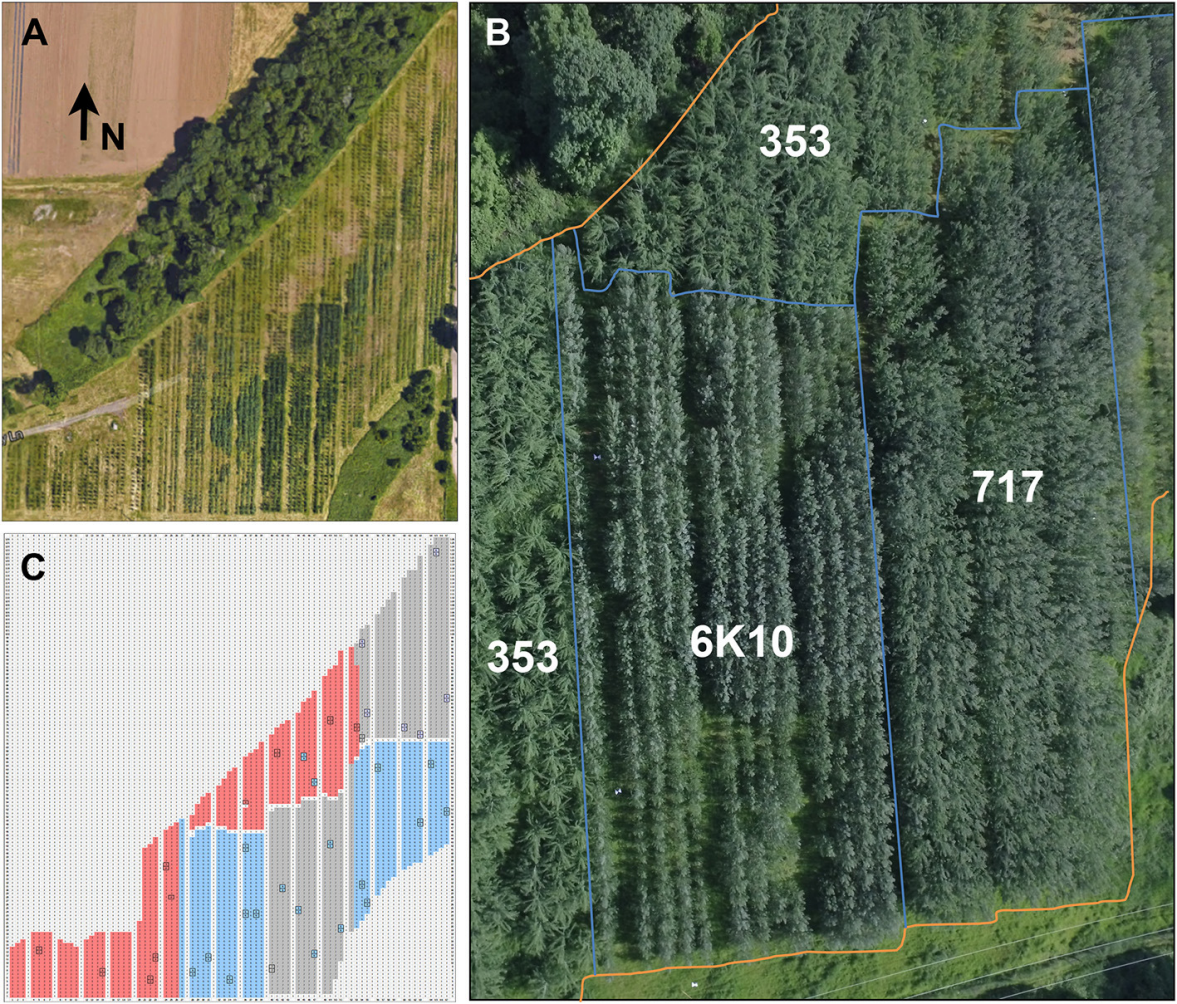
Plantation overview. **(A)** The plantation consisted of 3.6 hectares of hybrid poplar trees surrounded by greenbelt and agricultural areas. The entire plantation was enclosed in deer exclusion fencing. Arrow indicates the direction North (N). Note the variation in foliage color. **(B)** An overhead view of most of the plantation showing the locations of the blocks of each clone. Blue lines show clone boundaries, orange lines show plantation perimeter. **(C)** A graphical representation of the plantation showing the location of each clone. Locations without trees (wide rows, unplanted areas) are shown in white, male clone 353 in red, female clone 717 in gray, and female clone 6K10 in blue. Locations of non-transgenic control trees are boxed in black. Each clone was planted in two blocks, with trees from male clone 353 concentrated on the windward side of the plantation to serve as a pollen source for female clones 717 and 6K10.

Constructs were transformed into the three poplar clones and independent transformation events obtained. Vegetative propagation methods were used to obtain an average of four ramets (trees) from each transformation event. Events were planted in two-tree plots to make it easier to visually detect modifications to flowering and vegetative development. Each row-plot was planted at random in each of two blocks for the three poplar clones (they were separated into blocks due to their distinct rates of growth, and thus likely shade induced mortality prior to flowering) (Figure 3). A total of 3,315 trees (Table 3), including controls, were planted in approximately over 3.6 hectares. These included 1,112 trees of male clone 353, 1,254 trees of clone female 717, and 1,139 tree of female clone 6K10. The plantation was located in Western Oregon, a region characterized by a warm dry summer and cool wet winter. The trees were not protected from the elements and experienced a very hard freeze in 2014 and a usually hot dry summer in 2016. Tree survival was scored yearly; by the end of 2017 survival for all trees was 94.6% (as determined by number of trees currently alive versus number of trees planted). Male clone 353 had the lowest survival of 91.0%, female clone 717 had a survival rate of 94.1%, and female clone 6K10 the highest rate of survival at 98.6%.

**Table 3:** Construct names and observed outcomes.

**Construct name**	**Predicted outcome**	**Observed outcomes by clone**		
		**353**	**717**	**6K10**
Control	No change	Normal	Normal	Normal
AGL24-OE	Early flowering	Normal	Normal	Normal
AG-M2	Delayed flowering	Normal	Normal	Na
AG-M3	Delayed flowering	Normal	Normal	Normal
AP1-M2	Delayed flowering	**Delayed**	**Delayed**	Na
AP1-M3	Delayed flowering	**Delayed**	**Delayed**	**Delayed**
FT	Delayed flowering	Normal	Normal	Normal
SVP-OE	Delayed flowering	**Delayed**	**Delayed**	**Delayed**
AGL20	Delayed flowering	Normal	Normal	Normal
AGL24	Delayed flowering	Normal	Normal	Normal
FT:AGL20:FPF1	Delayed flowering	Normal	Normal	Na
FT:AGL20	Delayed flowering	Normal	Normal	Na
PFPFL1	Delayed flowering	Normal	Normal	Na
PFPFL2	Delayed flowering	Normal	Normal	Normal
PTAG	Sterile flowers	**Floral alterations**	Normal	**Female sterile**
MpTAG	Sterile flowers	Na	Na	**Female sterile**
PTAP:PTAG	Sterile flowers	Normal	Normal	**Floral alterations**
PTAP:PTLF	Sterile flowers	**Bisexual**	Normal	**Floral alterations**
PTAP	Sterile flowers	Normal	Normal	Normal
PTD	Sterile flowers	Normal	Normal	Normal
PTLF:PTAG	Sterile flowers	Normal	Normal	**Floral alterations**
Triple	Sterile flowers	Normal	Normal	Normal
PTLF	Sterile flowers	**Male sterile, bisexual, female**	Normal	**Female sterile**
PTLF+PTAG	Sterile flowers	**Male sterile, bisexual**	Normal	**Floral alterations**

*Flowers and annual onset of flowering from all constructs and clones were scored. Normal, normal form and onset; delayed, late onset; floral alterations, organ identity changes without loss of fertility; bisexual, male and female organs on male clone; female, female organs on male clone; sterile, loss of ovules or pollen; NA, not applicable as no events were planted for that construct and clone. Bold letters indicate modified floral phenotypes*.

**Figure 4:**
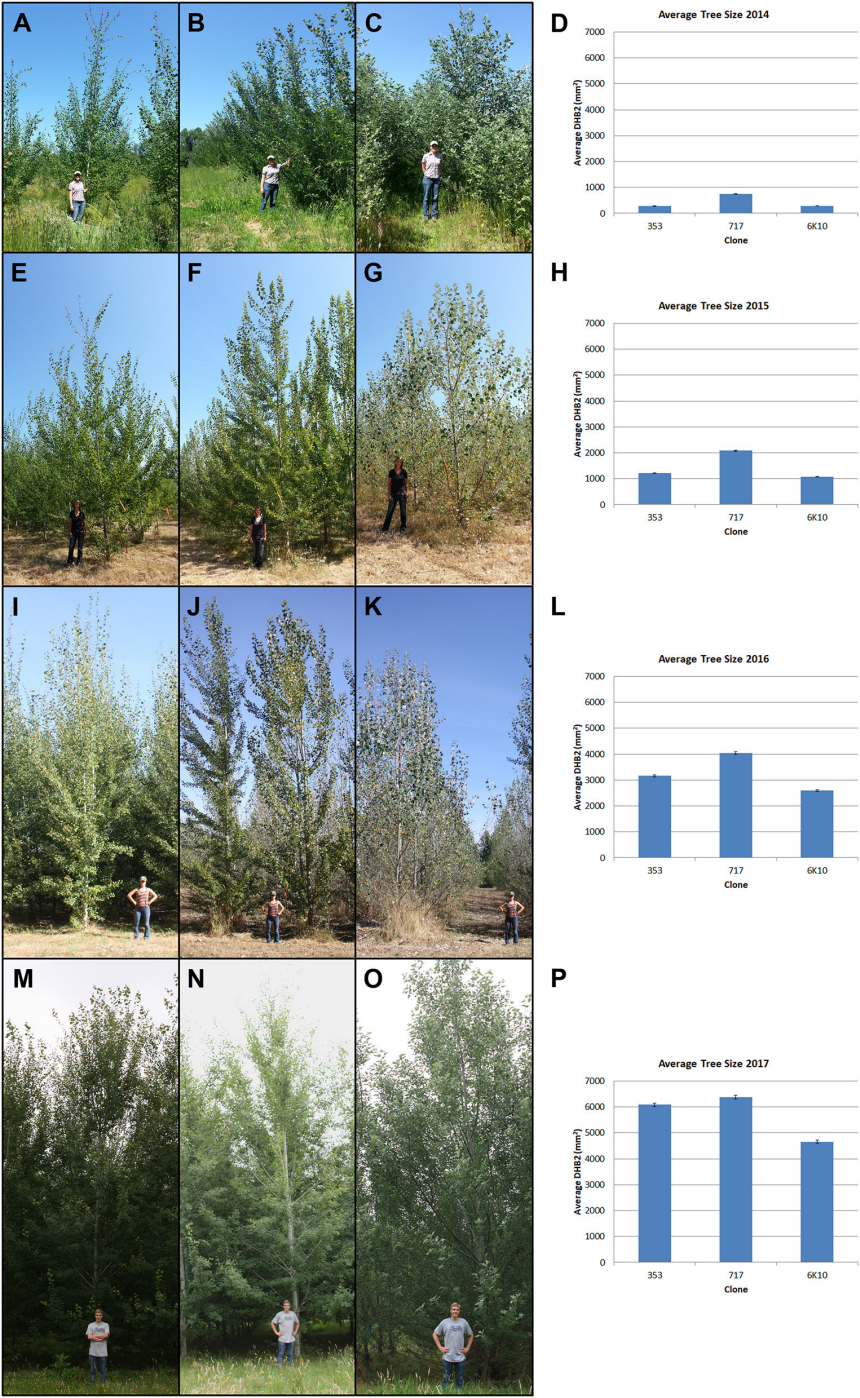
Stages of tree growth for the three tested clones. **(A–C)** Clones 353, 717 and 6K10 with field manager Kori Ault in June 2014. **(E–G)** Clones 353, 717 and 6K10 with field student Anna Magnuson in August 2015. **(I–K)** Clones 353, 717 and 6K10 with field student Lauren Yap in August 2016. **(M–O)** Clones 353, 717 and 6K10 with field student Thomas Howe in June 2017. Graphs show average tree size by clone, as determined by DBH^2^, in **(D)** 2014, **(H)** 2015, **(L)** 2016 and **(P)** 2017. Bars show standard error of the mean of all trees per clone.

Tree size was measured yearly for all trees in the plantation. Both trunk diameter at breast height (DBH) and overall tree height were measured until 2016 (when many trees outgrew the height pole); from 2016 onwards DBH was used for size measurements. All three clones generally grew well across the growing seasons (Figure 4). Analysis of tree size by clone and construct showed that in 2018 most events in each clone were performing well (Supplementary Figure 1). By the 2016 growing season most areas of the plantation were showing canopy closure, meaning that the branches of neighboring trees overlapped. Very soon after planting it became obvious that tree performance varied widely by location (Figure 3). Even in 2017 some low productivity areas still have bare ground visible, such as in the most northern block of male clone 353, indicating that even weeds do not grow well in these locations. Other regions had very large trees and extensive growth of all vegetation, making weed control a constant management challenge.

**Figure 5:**
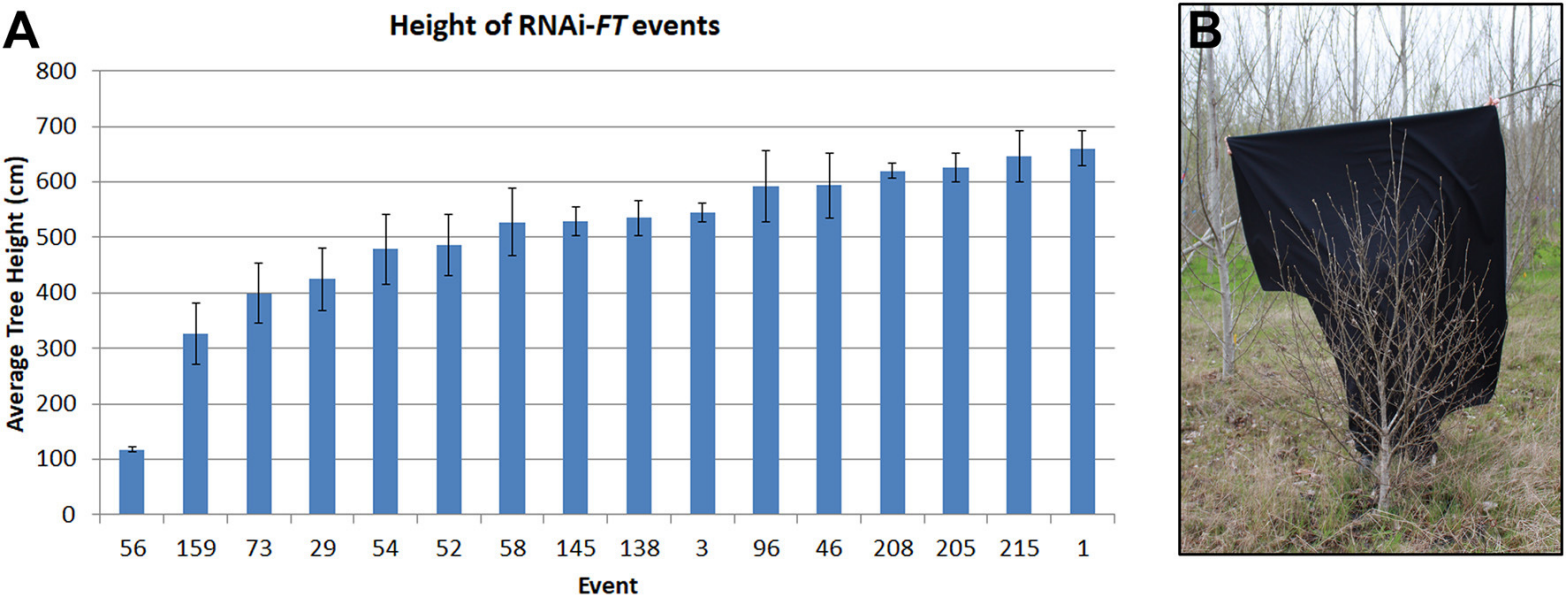
RNAi of *FT* genes led to some dwarf trees. **(A)** Average height of RNAi-*FT* events in female clone 717 as measured in 2015. Bars show average tree height for each event; standard error of the mean is shown. **(B)** Some RNAi-*FT* events showed greatly reduced vegetative growth, with a shorter height and copious branching, as compared to neighboring trees; event 56 from clone 717 is shown. Image from February 2017.

As the trees became larger differences in performance between neighboring construct pairs became increasingly obvious, indicative of construct and event differences. For example, it was noticed early on that some events from the RNAi-*FT* construct were very small (Figure 5), despite being located in areas of the plantation where neighboring trees grew well. In addition to their shorter height, these trees also had short internodes, giving them a bushy appearance. Similar results were observed for all three poplar. The RNAi-*FT* had been designed with the hope of obtaining delayed floral onset, without reductions in vegetative performance. When the work was initiated, the endogenous function of *Populus FT* homologs was unknown. While overexpression of either *PtFT1* or *PTFT2* could lead to early-onset of flowering (Bohlenius et al., 2006; Hsu et al., 2006) the genes have divergent functions, with *PtFT1* controlling the onset of flowering, and *PtFT2* controlling vegetative growth (Hsu et al., 2011). Given that *PtFT1* and *PtFT2* are 89.1% identical at the transcript level, it is very likely that both are being suppressed by the RNAi construct.

**Figure 6:**
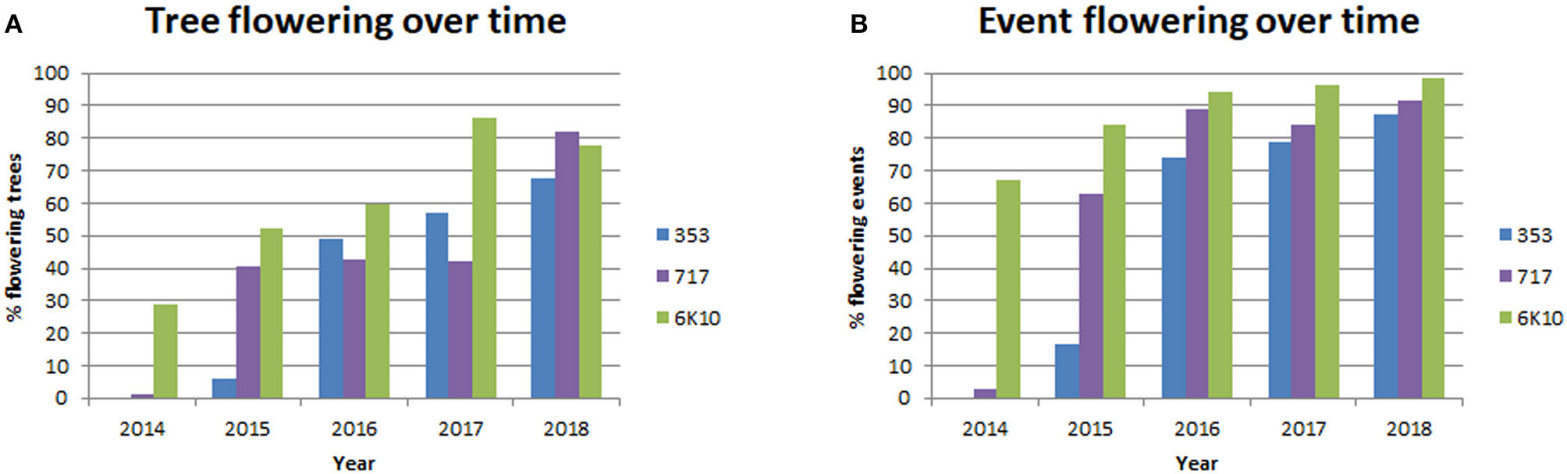
Tree flowering increased with time. Trees initiated flowering in 2014 and each tree was scored yearly for the presence or absence of floral buds. Events were considered flowering if at least one tree from the event had floral buds. Bars show the percentage of tree flowering **(A)** and the percentage of event flowering **(B)** for male clone 353 (blue bars), female clone 717 (purple bars) and female clone 6K10 (green bars).

A main goal of this study was to identify gene targets and methods (RNAi, DNM, overexpression) that would be useful for genetic containment by leading to prevention or long term delay in the onset of flowering. Trees were screened yearly for the presence of floral buds (before leaf flush), and dormant floral buds were first observed in January 2014 (Supplementary Figure 2). Each tree in the plantation was visually screened, and if at least one floral bud was observed then the tree was designed as flowering. If no floral buds were observed then the tree was designated as non-flowering. Colored flagging was used to mark flowering trees in the field; when floral buds flushed trees were re-evaluated for flowering as open flowers are larger and easier to identify than closed floral buds. Yearly floral scoring showed that both female clones started flowering in 2014, while male clone 353 started flowering in 2015 (Figure 6, Supplementary Table 2). Female clone 6K10 underwent noticeable increases in flowering each year, with 28.8% of trees flowering in 2014, which peaked at 86.4% flowering in 2017, with a small decrease to 77.8% in 2018. Male clone 353 also increased in flowering per year, with 6.0% flowering in 2015 and 67.6% flowering in 2018. Female clone 717 initiated flowering in 2014 with 1.0% of trees flowering, then showed 40.5% flowering in 2015, and by 2018 82.0% of trees flowered. The percentage of events flowering per year (events with at least one flowering tree were designated flowering) was generally similar to the percentage of trees flowering per year, with 87.2, 91.5, and 98.6 of events in clones 353, 717, and 6K10 flowering in 2018, respectively (Figure 6).

**Figure 7:**
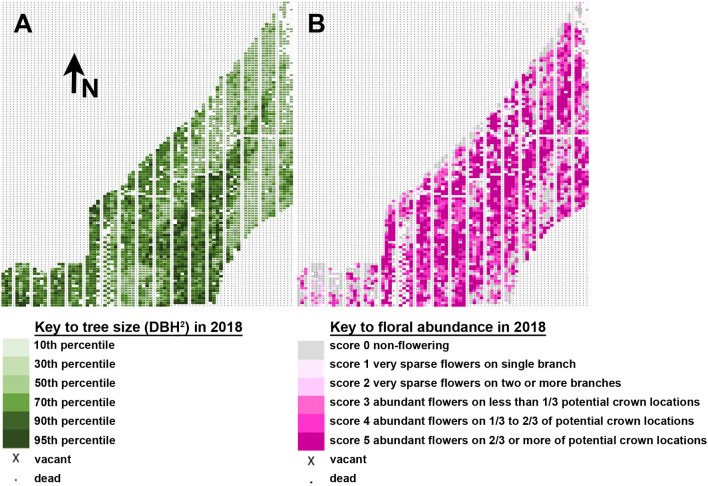
Tree location and size influenced flowering. **(A)** Map of tree flowering observed in 2018. Each rectangle indicates the position of one tree. Tree size is shown on a relative scale from smallest trees in the 10th percentile (pale green) to largest trees in the 95th percentile (dark green). Arrow indicates the direction North (N). **(B)** Map of floral abundance as observed in spring 2018. Floral abundance is shown on a relative intensity scale from no flowers (gray) to abundant flowers across 2/3 or more of potential crown locations (dark pink).

Tree flowering was impacted by tree location, which greatly affected rate of growth across the plantation. Mapping of tree size and tree flowering by location indicated a trend for larger trees tending to flower earlier and heavier (Figure 7), though there were also exceptions. A diagonal stripe of higher fertility soil runs southwest to northeast across the plantation, and thus had most of the larger and more intensely flowering trees. There were also regions of the plantation, however, such as the southwest corner, that showed good tree growth but little observed flowering, despite having a mix of constructs and events in the area. Other locations had smaller trees, such as the southeast region, but copious floral production. Our results showed that soil quality likely had complex effects on the onset of flowering beyond that due to growth rate alone.

**Figure 8:**
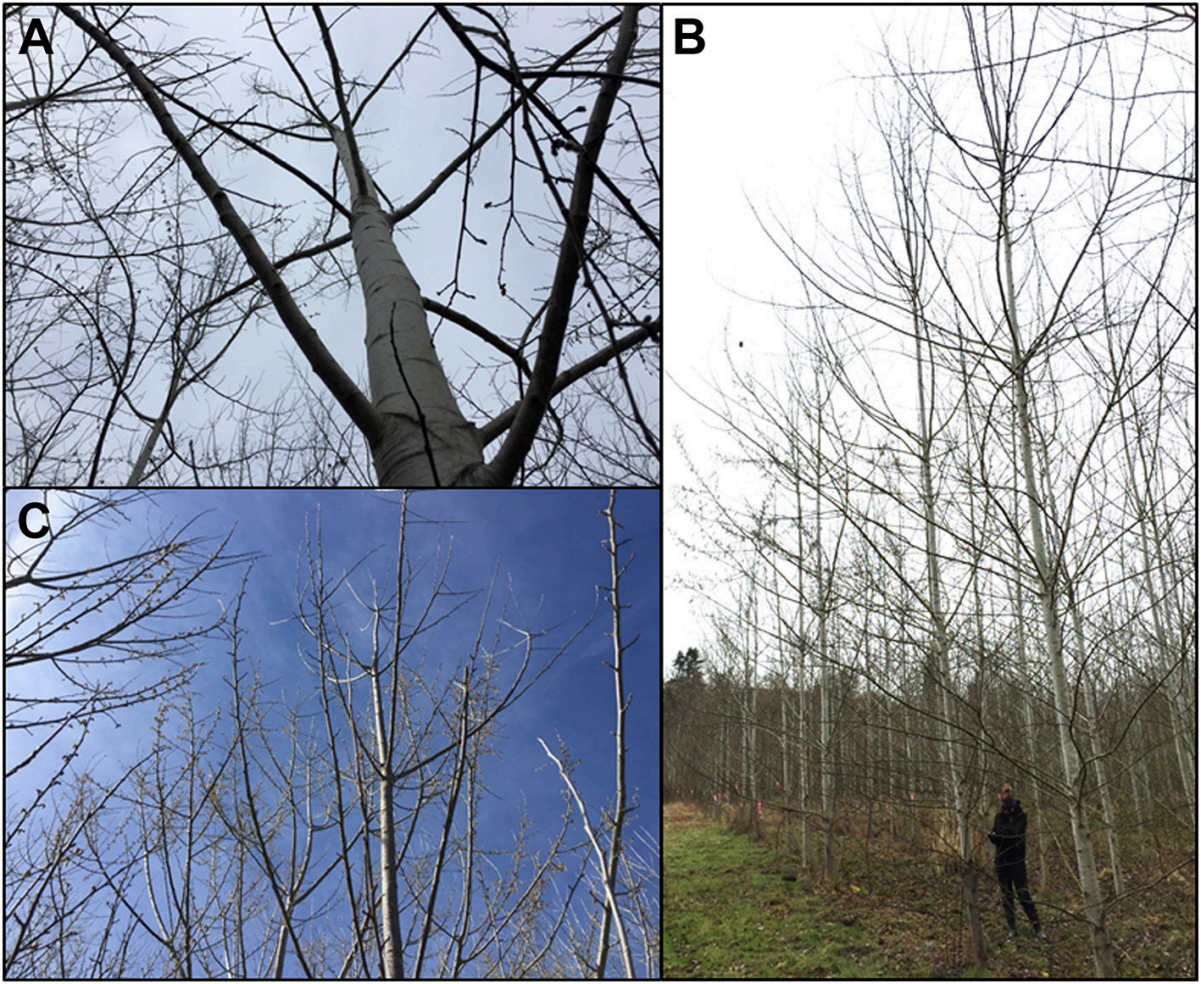
Overexpression of floral suppressor genes prevented or postponed the onset of flowering in poplar. An eight-year-old field trial (photos taken in March 2018) showed many examples of large trees from constructs and events with little or no flowering when nearby trees flowered heavily. **(A)** View up the trunk of a large non-flowering tree from RNAi of poplar AGL24 in male clone 353. **(B)** Overview of plantation row of female clone 6K10 with a pair of non-flowering ramets (foreground) expressing a DMN version based on the *A. thaliana AP1* gene (construct AP1-M3); neighboring flowering trees are visible further down the row. **(C)** A large non-flowering tree (center) from an SVP-OvExp event in female clone 6K10, surrounded by flowering trees.

**Figure 9:**
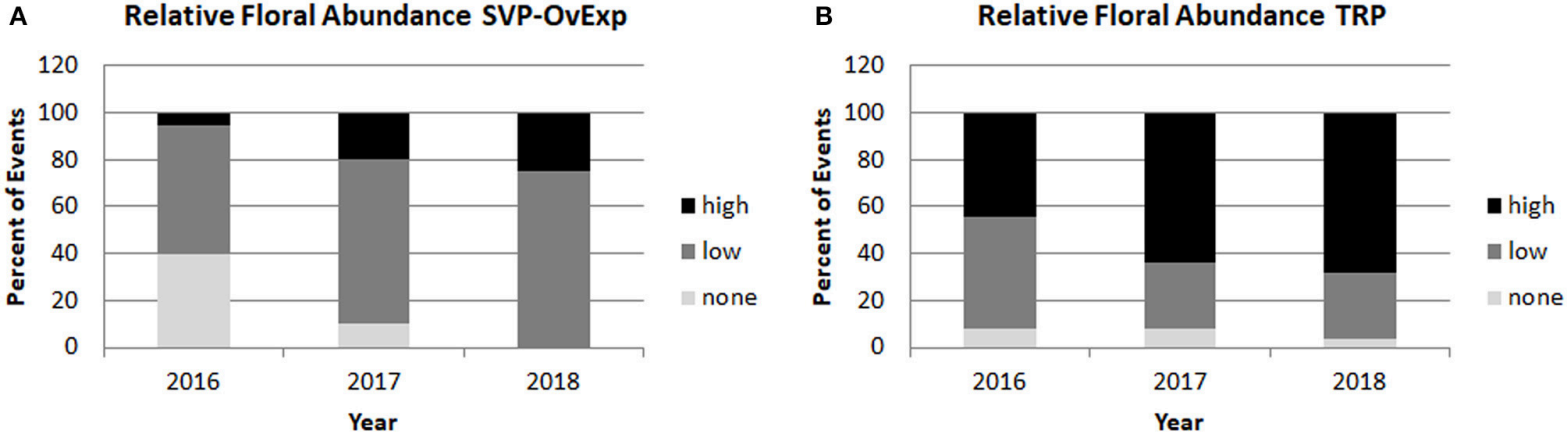
Overexpression of floral suppressor SVP led to reduced floral abundance across events and years. Relative floral abundance was scored yearly for all trees. Percentage of events with average floral scores of 0 (corresponding to no floral buds) were categorized as none, events with average floral scores of less than 3 (meaning less than 1/3 of the crown had copious floral buds) were categorized as low, and events with average floral scores of 3 or higher (meaning at least 1/3 to the entire crown had copious floral buds) were categorized as high. Yearly floral abundance data from clone 6K10 events transformed with **(A)** and SVP-OvExp construct or **(B)** an RNAi construct targeting the *LFY, AG*. and *AP1* genes that did not affect floral onset are shown.

Starting in 2016 relative floral abundance was scored for each tree, ranging from no flowers (score of 0), to copious flowers across the entire canopy (score of 5); the full scoring system is given in Figure 7. Analysis of floral abundance by construct allowed for the identification of constructs and events with reduced flowering. For example, constructs overexpressing *SHORT VEGETATIVE PHASE* (*SVP*) or a dominant negative version of the *A. thaliana APETALA1* gene (*AP1*), or RNAi- suppressing the *AGL24* gene, had events with large trees that flowered very little or not at all, even when neighboring trees flowered heavily (Figure 8). Analysis of the relative floral abundance across SVP-OvExp events in clone 6K10, our poplar clone with the highest percentage of flowering events (Figure 6), showed that most of these events had little flowering, even in 2018 when essentially all events from controls and normal flowering-onset constructs had flowered (Figure 9). By contrast, events from the TRP construct, which was designed to disrupt floral structure not onset, had very abundant flowering per event.

A second main goal for this study was to identify constructs that led to altered (ideally sterile) inflorescences or floral organs. As the flowers tended to open more or less simultaneously per clone and were only open for a brief amount of time in the field during the rainy and cold Oregon winter, branches with dormant floral buds were collected during winter and flushed in a warm laboratory for initial screening of floral form (Supplementary Figure 2). Floral buds are larger than vegetative buds and can be easily recognized in the field. The large majority of flowers observed in the lab were similar to those of control trees. However, some events from RNAi constructs targeting the *LEAFY* (*LFY*) and *AGAMOUS* (*AG*) genes had noticeably different floral forms. Some RNAi-*LFY* events had female flowers with no externally visible carpels and were determined to be sterile (Klocko et al., 2016). Select RNAi-*AG* events had female catkins which opened early and appeared to be larger than control catkins, some of which were also determined to be sterile. Data from the lab were then used to identify constructs and events of interest for observation in the field.

**Figure 10:**
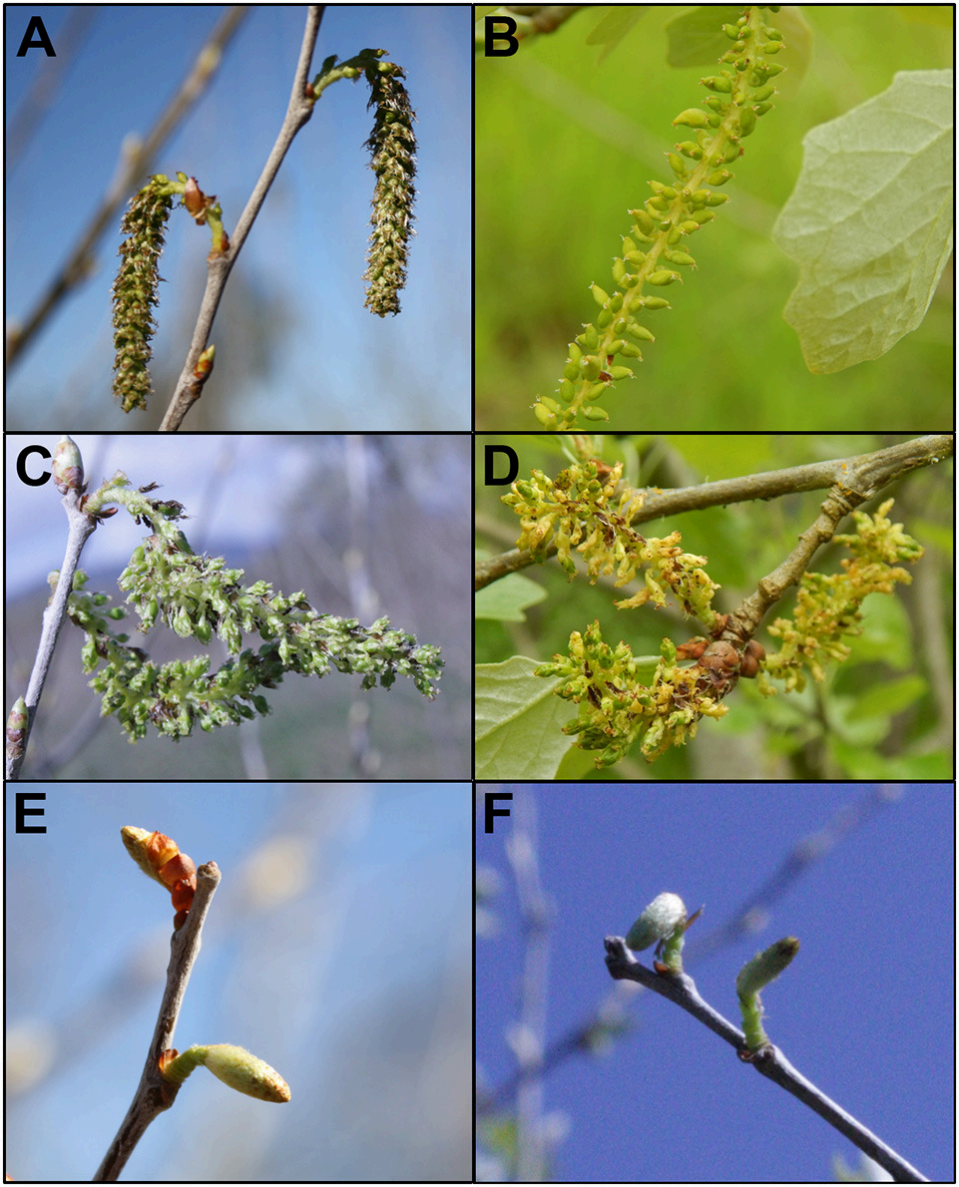
Events with strong floral phenotypes were stable across flowering seasons. Flowers from wild type female 6K10 showed similar catkin formation in **(A)** 2014 and **(B)** 2017. Flowers from RNAi-AG (mar) event 165 showed catkins with replicated carpels in **(C)** 2014 and **(D)** 2017. Flowers from RNAi-LFY event 139 showed small catkins with no externally-visible carpels in **(E)** 2014 and **(F)** 2015.

**Figure 11:**
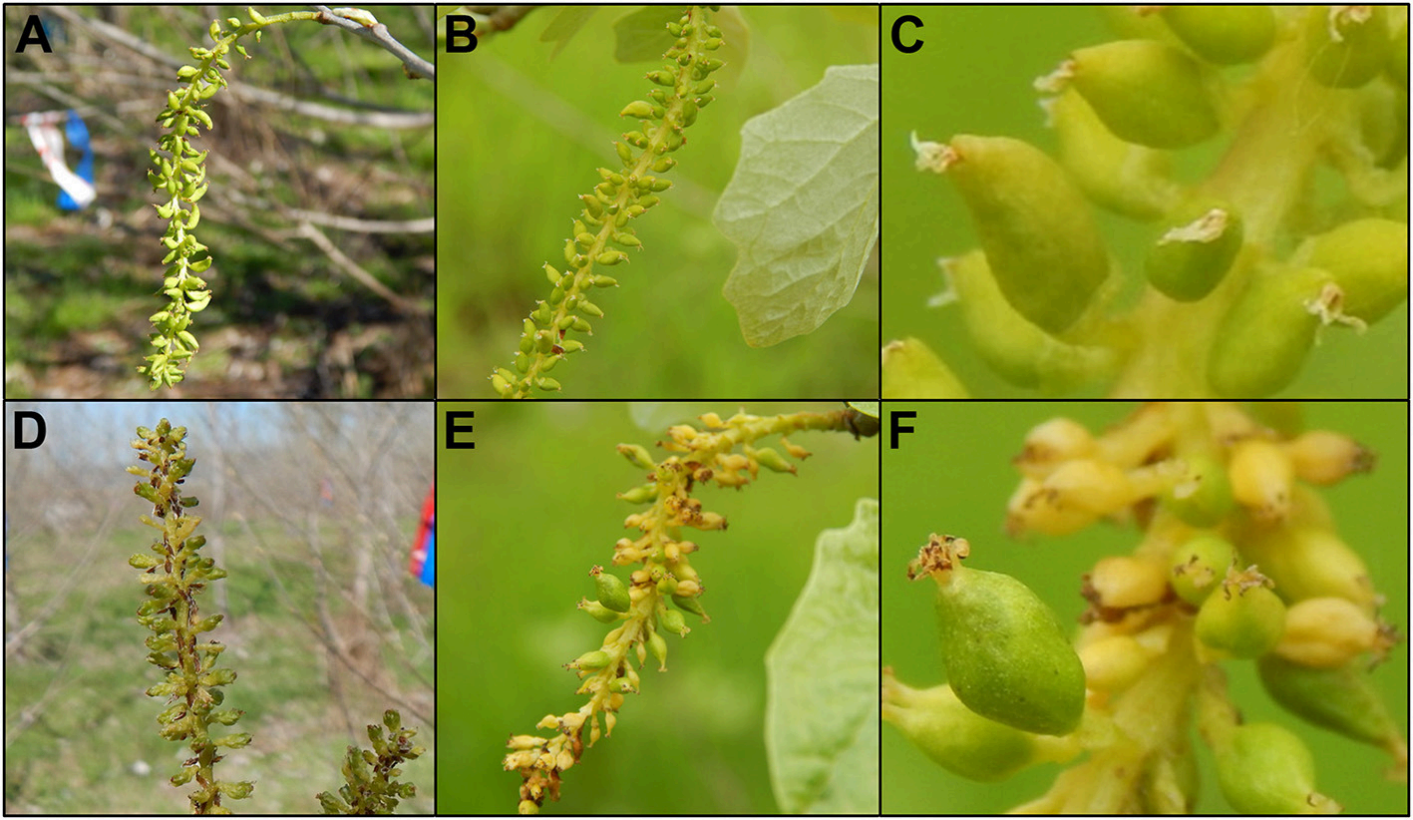
Trees with intermediate levels of RNAi suppression had catkins with variable phenotypes. Flowers from 6K10 control trees in **(A)** 2016, **(B)** 2017 showed well-formed carpels. **(C)** Carpels were of uniform appearance across the catkin. Upright catkins from RNAi-*AG* (mar) event 119 from **(D)** 2016 and **(E)** dangling catkins from the same event in 2017. **(F)** Carpels from these flowers had non-uniform sizes, colors or shapes.

**Figure 12:**
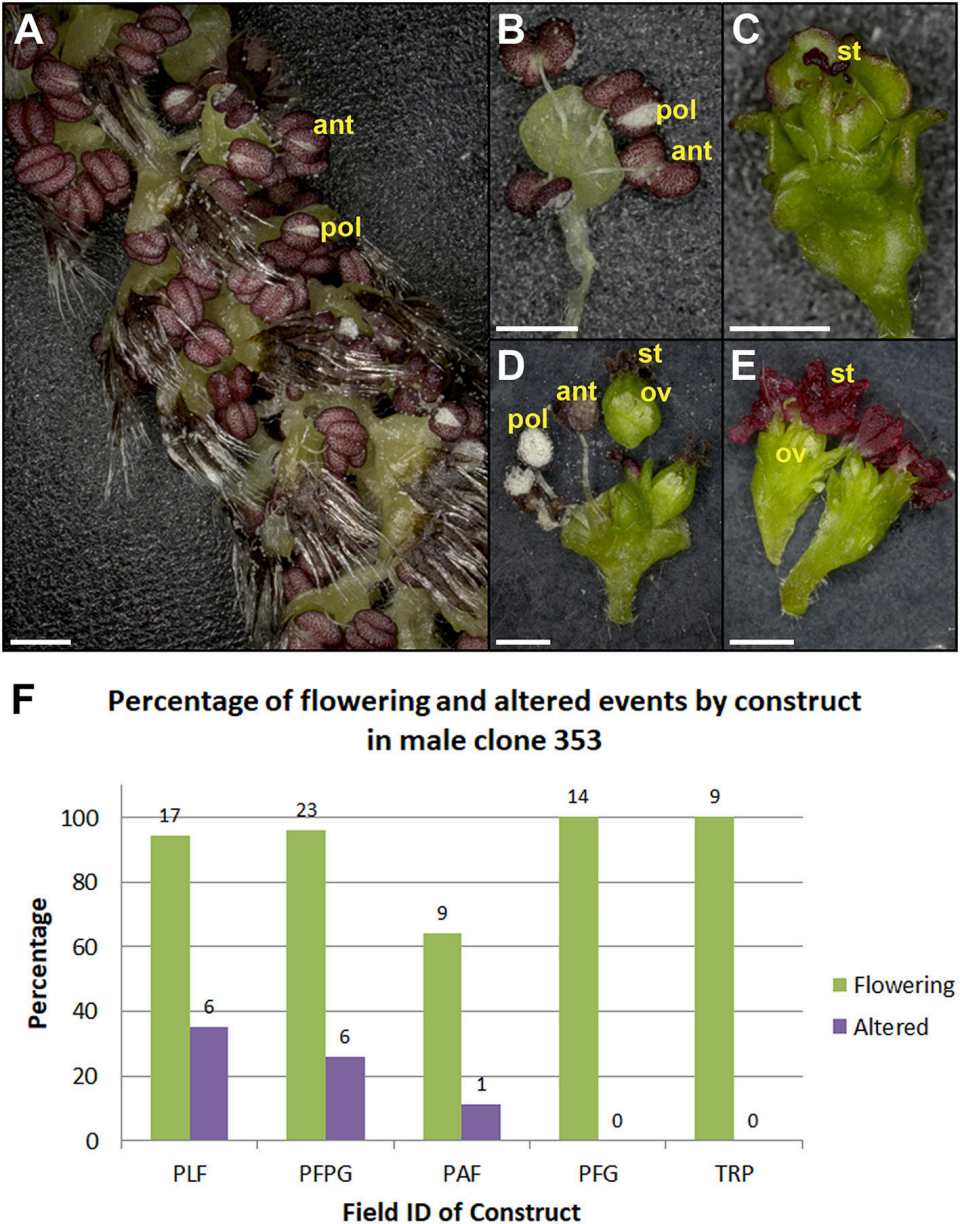
Male clone 353 had variable floral phenotypes from constructs giving strong female sterility. Normal looking **(A)** catkin and **(B)** flower from RNAi-LFY/AG event. **(C)** Sterile male flower from RNAi-LFY/AG event. **(D)** Dissected bisexual flowers with ovules from RNAi-LFY event. The carpel in D was sectioned and placed above the rest of the flower to better display the ovules. **(E)** Dissected female flowers with ovules from RNAi-LFY event. ant, anther; pol, pollen grains; st, stigma; ov, ovule. Scale bars denote 1 mm. **(F)** Graph of flowering events and floral morphology in male clone 353 from 2018. Bars show the percentage of all flowering events (green bars) and events with floral alterations (purple bars) from RNAi-*LFY* and related constructs. PLF, RNAi-*LFY*; PFPG, RNAi-*LFY*+RNAi-*AG*; PAF, RNAi-*LFY*:*AP1*; PFG, RNAi-*LFY:AG*; TRP, RNAi-*LFY*:*AP1*:*AG*. Numbers above bars denote the number of events.

Observation of floral form in the field showed that events had similar phenotypes in the field as they did in the lab. In addition, events with strong floral modifications had stable phenotypes across growing seasons (Figure 10). Other events from the RNAi-*AG* constructs had intermediate floral phenotypes (Figure 11), and flowers from these events continued to show floral variability, such as mixtures of fertile and sterile capsules on single catkins (green vs. yellow capsules, Figures 11E,F). Variation was also observed between male and female clones transformed with the same construct. The same RNAi-*LFY* construct which led to strong female sterile phenotypes gave rise to bisexual or female flowers in male clone 353 (Figure 12). Two other constructs targeting *LFY*, either singly or together with the *AG* or *AP1* genes, also led to floral alterations in this clone. Overall, 11 constructs of the 23 tested led to alterations in floral morphology or floral timing in at least one clone (Table 3).

Part of the permit requirements for allowing flowering at the field site was a yearly analysis of seed production, seed viability, and frequent screening for the establishment of seedlings in and around the field location (leaf morphology is distinct from wild poplars for the tested clones). Each year catkins from all flowering female clones and constructs were sampled and screened for the presence of seeds, and seeds tested for viability in lab conditions (Supplementary Tables 3, 4). From 2014 through 2017 a total of 300 seeds from female clone 6K10 were found, and a total of 140 seeds from female clone 717 were found. All seeds found were tested for viability by germination testing. For female clone 6K10 the percent germination ranged from 0% of the 10 seeds found in 2014 to 21.7% of the 106 seeds found in 2016, with an overall germination rate of 13.7% for all seeds found in all years. For female clone 717 the percent germination ranged from 5.6% of the 18 seeds found in 2017 to 50.0% of the 2 seeds found in 2014, with an overall germination rate of 28.6% for all seeds found in all years. In addition to laboratory seed testing, the field site itself and the surrounding perimeter were checked for seedlings. No transgenic tree-derived seedlings were identified in the field site or the surrounding perimeter.

Poplar trees can also spread by means of vegetative propagation. Therefore, the site and surrounding perimeter were regularly monitored for the presence of vegetative sprouts, termed suckers. All planted trees had a shade cloth and metal field tag and were planted in a gridded spacing, allowing for the identification of any unplanted poplar shoots. Such vegetative suckers were rare, and were killed when found by spraying them with herbicide, uprooting the stem, and burning the plant material. Low numbers of suckers were found in the field site itself, and all were devitalized shortly after discovery.

## Discussion

The goal of this field trial was to analyze the effectiveness of 23 different genetic constructs and 15 target genes for obtaining delayed or modified flowering in poplar, hopefully enabling a high level of genetic containment. Ideally, such trees would have either delayed floral onset or reduced floral fertility without negative impacts on vegetative performance. It was clear that tree growth was uneven across the field site (Figures 3, 7). The site used for the field plantings was previously used for residential and agricultural purposes, and there may be foundation remains, gravel, soils of varying past fertilization, and compacted soil or buried debris, any of which could impact tree performance. The site was also characterized by strips of variable natural soils as a result of past floods and variable sedimentation by the nearby Willamette River. This variation in growth complicated the interpretation of vegetative performance. However, when averaged over the dozens to thousands of trees studied it was clear that the large majority of trees grew well without regard to construct (Supplementary Figure 1), and by 2016 most were of a substantial size (Figure 4).

Yearly scoring of the flowering which started in 2014 provided us with five years of floral onset data for analysis (Figure 6). All trees were planted at the same time and were the same age. The three clones varied in the timing and abundance of floral onset, with female clone 6K10 showing the earliest and highest initial percent of flowering, and male clone 353 showing the latest flowering (Figure 6). For all clones, the percent of flowering tended to increase with tree age, as would be expected. Ideally, all trees from a given clone would have relatively synchronized flowering, allowing for easy identification in alterations of floral timing. However, we found that tree location greatly impacted tree performance. For example, while female clone 6K10 flowered the most abundantly of all three clones (Figure 6), portions of one block had very low numbers of flowering trees, likely due to variability in the soil quality at that position (Figure 7).

We also noticed that the amount of flowers present on each tree varied greatly. Starting in 2016 we scored the relative abundance of flowers present on each tree. At this time about half of the trees, across all three clones, were flowering (Figure 6). The variation in tree flowering across the site (Figure 7) added to the complexity of determining which constructs and events were leading to delayed flowering or decreased floral abundance. Therefore, we focused on identifying constructs and events with low rates of flowering, or low floral abundance, particularly if trees from such events were located next to other trees with abundant flowering. We found that three constructs most clearly led to delays in floral onset or a decrease in overall floral abundance (Table 3). Importantly, based on visual inspection these trees had normal productivity. We found that overexpression of *SVP*, or DNM versions of *A. thaliana AP1* or RNAi of the *AGL24* gene, led to trees that had reduced floral abundance or flowered years later than neighboring trees (Figures 8, 9).

Many of the constructs studied were designed to allow flowering, but to alter floral structure to impair formation of pollen or seeds (Table 2). We found that targeting of the *LFY* or *AG* genes led to altered, potentially sterile flowers in female clone 6K10 (Figure 10, Klocko et al., 2016; Lu et al., 2018). When the floral alterations were strong and the floral phenotype uniform, these traits were stable across flowering seasons (Figure 10), while intermediate traits continued to show variability (Figure 11). Events with strong and stable traits would be the most useful for achieving reliable containment. However, it is estimated that even imperfect sterility would greatly reduce gene flow from GE plantations (DiFazio et al., 2012).

Another key finding from this work was the challenge of predicting outcomes across clones. Ideally, each construct would have comparable impacts in each genetic background. We did find that some constructs, such as SVP-OvExp (Figure 8), had similar phenotypic outcomes across clones. However, that was not always the case. The same RNAi-*LFY* construct which led to strong female sterility in female clone 6K10 (Figure 10) had variable floral phenotypes in male clone 353 (Figure 12). Some RNAi-*LFY* events in this male clone had bisexual flowers, or even female flowers. This sort of floral gender change phenotype was previously observed on female clone 717 trees overexpressing poplar *LFY* (Rottmann et al., 2000).

As part of our regulatory permit, we monitored the spread of the trees locally by vegetative shoots, and by seed formation and seedling establishment. Such data are informative regarding the actual risks of spread by vegetative means or sexual reproduction. We did find a small number of vegetative sprouts very close to plantation trees; these were easily killed by herbicide sprays and uprooting the stems. Regular mowing for weed control was likely a contributing factor to the low observed numbers of suckers, as they would be cut off very low to the ground. Such practices are common in managed tree plantations. Yearly surveys for seeds and seedlings showed that while seeds were formed and some were viable under lab conditions (Supplementary Tables 3, 4), no seedlings were found at the field site. Thus, the possibility of spread into neighboring wild populations by seed dispersal and seedling establishment is very low. This is not surprising as it is well known that poplars require special conditions for establishment due to their very small seeds; this includes moist soils during early stages of growth that are free from competition from fast growing weeds (DiFazio et al., 2012). The continuous grass and weed cover around the plantation, and nearby closed forest or annual agriculture, did not provide such permissive conditions.

For genetic containment systems that are acceptable in commercial forestry, it is essential that the genes employed do not adversely affect vegetative growth. Although most of the tested constructs had no detectable effects on vegetative growth, we found that some RNAi-*FT* events were dwarfed in size and had altered vegetative form (Figure 5). At the time the work was initiated, it was not known that the two poplar *FT* genes had divergent functions, or indeed that there were two *FT* genes. The small size and altered form of some RNAi-*FT* trees indicate that the *FT2* was likely suppressed, and this gene is important for vegetative performance (Hsu et al., 2011).

One challenge for this trial was managing the large number of trees that needed to be monitored over several years of study. This is a result of the variability in RNAi suppression or overexpression among gene insertion events (requiring as many events as possible to see a range of effects), the desire to study male and female flowers, the inclusion of normal and early flowering poplar clones, environmental variation in the plantation as discussed above, and the multiple year delay until onset of flowering in these trees. In total we tested 948 independent transformation events over 8 growing seasons (Supplementary Table 1). As we also sought to obtain replicate trees from each event, the numbers of trees needed for analysis was multiplied about four, for a total of 3,315 trees. The variability of RNAi effectiveness among events also means that some constructs could have led to sterile or delayed flowering had additional events been analyzed. For example, no events with altered flowers were observed for trees transformed with the TRP construct, which was designed to suppress the *LFY AG* and *AP1* genes simultaneously from a single hairpin (Table 2). However, obtaining strong suppression for all five targets (both *AG* and *AP1* are duplicated in poplar genome) might have required that we test many dozens or even hundreds of events; this was beyond our capability and resources. For goals such as multiple gene knockouts, gene editing technology, especially CRISPR, should be far more efficient, and knock-outs can be identified in the laboratory and only a small sample propagated and planted in the field. They are also likely to be far more stable than gene suppression or overexpression technologies, enabling confident genetic containment and thus improving public acceptability and simplifying regulatory decisions.

Our data show that suppression of the *LFY* and *AG* genes with other RNAi constructs led to floral alterations (Figures 10–12, Table 3), but for some reason combinatorial constructs were unsuccessful in this study. It is likely that the type of RNAi construct affects the rate of multiple gene suppression. For example, we tested two different constructs to simultaneously suppress the *LFY* and *AG* genes (Table 2). The PFPG construct had two hairpins, one for *LFY* and one for the *AG* genes, and the PFG construct had a single hairpin containing both inverted repeats. The two hairpin construct led to floral alterations and the single hairpin construct resulted in normal flowers in male clone 353 (Figure 12). The two hairpin PFPG construct also led to floral alterations in female clone 6K10, but the single hairpin also did (Table 2).

A second challenge from this trial was related to the sheer size of the site, the number of trees, and multiple-year duration of the trial. In addition to the expected challenges of weed control and irrigation, damage to trees from biotic sources was a persistent challenge. Deer were found to be particularly tricky adversaries, capable of squeezing under fence lines. With over 3.5 hectares of trees to hide in and no predators, our trial also provided the deer with an excellent source of shelter and food. We also found that shade cloths placed under each tree for weed suppression were utilized by rodents for cover, and often damaged trees by girdling (Figure 1). Human vandals were a more worrisome but thankfully less frequent source of damage; the most recent harm to our trees occurred in 2001 (Figure 1), and no damage has occurred since.

The large size and delayed flowering of clones 353 and 717 made floral collections challenging. Dormant floral bud sampling in 2016 and 2017 required a pole pruner that included a set of clippers located at the end of an extendable pole. Tree size will also present a continuing challenge at the time of trial termination. Once a field trial is complete, all trees must be killed and the area monitored until no new sprouts have been observed for two full years. This task can be quite daunting for poplar trees, which are extremely good at re-sprouting from their roots, even after herbicide treatment of stumps or sprouts. Carefully chosen herbicides, applied at the optimal times of year, and some years of retreatment of sprouts, are likely to be needed based on our past experience.

Obtaining and maintain regulatory approvals for a flowering field trial of trees is difficult; most researchers do not attempt it. However, as modification of fertility was the point of the study, there were far too many large trees to consider bagging of all flowers, and performance of containment technology under natural plantation conditions was our goal, there was no choice but to seek approval for normal flowering. Fortunately, the use of aspen/white poplar clones that are not compatible with native cottonwood *Populus trichocarpa*, the very specialized establishment needs for poplar, and the innate biosafety of tree sterility traits (and potential containment benefits in the future) prompted USDA to agree that our field trial was safe to conduct. The need for *any* containment for a field trial of containment genes seems absurd to us, but is the product of a system that is focused on the method of modification and the vectors and genes used, not the novelty and risks nor the potential benefits, of the resulting traits. However, obstacles to field trials of GE trees are much more severe in many other parts of the world (Viswanath et al., 2012); we are fortunate to have a workable, science informed system in the USA. Nonetheless, we devoted substantial effort to producing numerous permit applications, reports, and undergoing inspections that are very difficult for most academic and public sector laboratories to afford.

In sum, we obtained valuable lessons about gene function, stability of trait expression, and containment options from our multiple-year field trial. All of these lessons support the finding that GE methods of genetic containment, specifically RNAi and overexpression, can be very effective and reliable for reducing risks of gene flow. Our results have identified several genes and types of genetic modifications that warrant further study given our findings. Future work will hopefully include a larger number of years that more closely approximate the commercial lifetime of plantation tree varieties, and examination of larger numbers of insertion events, especially for the RNAi constructs. The *AG* and *LFY* genes, in particular, appear to be very promising targets for bisexual sterility without obvious impacts on vegetative development; however, their impacts and performance in male clones is unclear, perhaps due to a lower rate of RNAi suppression in the male clone 353-53. The targeting of both of these genes with CRISPR is expected to be feasible and highly successful, establishing whether gene knock-down would indeed be a universal containment technology in poplar. Likewise, promoter editing of the *SVP* and other floral-onset suppressive genes might be superior to generic overexpression, and highly successful means for maintaining trees in a juvenile state to promote rapid growth and avoid flowering. The growing genomic and molecular knowledge of trees, combined with the precision of gene editing, suggest that many new and more powerful genetic innovations are just around the corner.

## Materials and methods

### Construct assembly

RNAi constructs were produced based on *Populus* sequences available at the time, which included partial to full-length cDNAs and the initial *P. trichocarpa* genome release. Gene fragments (Supplementary File 1) were cloned in the sense and antisense directions into the pHannibal vector (Wesley et al., 2001) creating a hairpin, prior to subcloning into the binary vector pART27. Hairpin expression was controlled by the *Cauliflower mosaic virus* 35S promoter, and the *Agrobacterium* tumefaciens octopine synthase (OCS) terminator. For RNAi constructs targeting unrelated genes, fragments of the targeted genes were first assembled in pBluescriptKS and the chimeric fragment then used to generate an RNAi transgene as described above. For the mPTAG vector, the RNAi transgene was inserted into the Not1 site of pG3KM (Li et al., 2008) and then the region between the TDNA borders excised with Acs1 and inserted into a modified pART27 vector (pART27A) where the TDNA region between the Not1 sites had been removed and replaced with an Acs1 linker. Dominant negative (DMN) constructs were alterations of the MADS-domain sequence based on previously described changes (Jeon et al., 2000). The M2 mutation of *AGAMOUS (AG)* and *APETALA1 (AP1)* was alteration of amino acids 30 and 31 from KK to EE, the M3 mutation of *AP1* and *AG* was alteration of amino acids 24 and 25 from RR to LE. The DNM transgenes were controlled by the double enhancer 35S promoter and the *Pisum satvia* E9 terminator. The DNM expression cassettes were assembled in pG3K (Li et al., 2008) and then the DNM and selectable marker transgenes were excised as a single fragment by Acs1 digestion and inserted into pART27A. Overexpression constructs were assembled in pCAPO, which is identical to the previously described pCAPT (Filichkin et al., 2006) except that the antisense fragment of the OCS terminator and PIV2 intron are absent.

### Plant transformation and field planting

Constructs were transformed into the three poplar clones using standard transformation methods (Filichkin et al., 2006). Tree propagation and field design were previously described (Klocko et al., 2016). In brief, rooted trees were planted in 6 blocks, such that each clone was present in two blocks. Pairs of trees from each transformation event were randomized in that block. Spacing between rows was 2.29 m, with a larger space of 6.10 m after every four rows to allow for vehicle access. Shade cloth was placed under each tree to aid in weed suppression, and each tree was labeled with a metal tag indicating the clone, construct, event and ramet. The field site was drip irrigated the first two summers (2011 and 2012) then discontinued as trees were well established. Weeds were controlled by mowing between rows and using a rotary motorized “weed-wacker” between trees.

### Tree survival and vegetative performance

Tree survival was scored each year at the time of vegetative bud flush. Tree size was measured by total height of the stem, and by stem diameter at breast height (DBH), a distance of 137 cm above ground level. Representative stands of each clone were imaged in the summer using a Canon Rebel XSI digital camera as a record of tree size. In spring 2017 an unmanned aerial vehicle (a drone) was used to obtain overhead images of the entire plantation.

### Floral scoring and indoor analysis of floral form

All trees were scored yearly in January and February for the presence or absence of dormant floral buds. Trees with at least one floral bud were designated as flowering. Trees with at least four branches with one or more buds were sampled by collecting small branch cuttings for floral analysis in the lab. Once flowers flushed in the field trees were rescreened to account for any floral buds missed in the initial survey. Collected twigs were stored at 4 degrees until they were analyzed in batches by clone and construct. Indoor flush was carried out by cutting off the ends of the twigs at a 45° angle and immediately placing the cut ends in cups of water. The plastic cups were inside a plastic bin lined with damp paper towels. Once all twigs were in water the entire bin was tented with a plastic bag to maintain high humidity, cut pieces of bamboo located in each corner of the tub kept the plastic from touching the branches. Branches were incubated at room temperature until most branches had enlarged catkins, about 5 days. Flushed twigs were photographed using a Canon Rebel XSI digital camera. Floral form was initially analyzed in the lab before buds flushed in the field.

### Scoring relative floral abundance

Starting in 2016 a floral abundance score was used as a means to categorize relative floral abundance. The entire crown of the tree was surveyed by two researchers, one on the east side of the tree and the other on the west side of the tree. Trees with no flowers were scored 0, trees with very sparse flowers on a single branch were scored 1, trees with very sparse flowers on two or more branches were scored a 2, trees with abundant flowers on less than 1/3 of potential crown locations were scored a 3, trees with abundant flowers on ½ to 2/3 of potential crown locations were scored a 4, trees with abundant flowers on 2/3 or more of the potential crown locations were scored a 5.

### Field analysis of floral form microscopy keyence digital microscope

Flowers that flushed in field conditions were photographed in the field using a Canon Rebel XSI digital camera. Selected flowers were collected, bagged and placed at 4 degrees. These flowers were imaged using a Keyence digital microscope VHX-6000.

### Catkin collection and seed presence and viability analysis

Starting in 2014, female catkins were collected from female clones 6K10 and 717. Trees were sampled such that catkins from at least two events (if available) were obtained from each construct and clone that flowered in that year. Catkins were collected into small paper envelopes, which were closed in the field then opened in the lab to allow catkins to dry, causing the release of cotton and seeds. Dry catkins were screened for seeds; any potential seeds were removed with tweezers and placed into 1.5 ml tubes until all catkins were screened. Seeds were counted then placed onto damp filter paper in 100 ml petri dishes. Dishes were sealed with parafilm to prevent moisture loss and incubated on the lab bench for 7 days. The number of germinated seeds was counted and tallied. Seeds were scored as germinated by the emergence of a root at least as long as the seed.

## Author contributions

AK, AB, and SS wrote the article. AB selected target genes and designed genetic constructs. AK, HL, and SS designed experiments. AK, HL, AM, SS, and CM collected data. AK, HL, AM, and CM analyzed data. Datasets are available on request.

### Conflict of interest statement

SS has directed a university and industry funded research consortium (TBGRC) based at Oregon State University for more than two decades that contributes to funding of research in his laboratory. Its work is directed at producing solutions to the problems of gene dispersal from genetically engineered and exotic trees. The remaining authors declare that the research was conducted in the absence of any commercial or financial relationships that could be construed as a potential conflict of interest.

## Supplementary material

Supplementary Figure 1 Trees from most constructs performed well in all three clones. Average tree size (DBH^2^) of all trees was calculated from measurements collected in early 2018. Graphs show average size of all trees per construct for **(A)** clone 353, **(B)** clone 717 and **(C)** clone 6K10. Note that clone 717 had a single non-transgenic control tree (CTR) which grew poorly. Bars show construct averages across all trees; standard error of the mean is shown.

Supplementary Figure 2 Dormant floral buds were flushed in the lab for initial floral classification. **(A)** Trees from female clone 6K10 in January 2015, trees with floral buds have blue flagging, trees with buds collected for indoor analysis have an additional red flag. **(B)** Small twig cuttings with dormant floral (fl) and vegetative (veg) buds. **(C)** Flushed control catkins, **(D)** flushed normal RNAi-LFY catkins, **(E)** RNAi-LFY twigs with very small catkins, **(F)** RNAi-AG (mar) twigs with enlarged catkins.

Supplementary Table 1 Numbers of trees planted and survival to date by clone and construct. Trees were first planted in 2011 and survival monitored yearly. Current numbers of surviving trees are from the 2017 spring bud flush. Event refers to individual transgenic occurrences; ramets are individual trees, each field ID refers to a unique genetic construct (see Table 2).

Supplementary Table 2 Flowering events by clone, construct and year. Tree flowering was monitored yearly, events with at least one flowering tree were considered flowering. NA for flowering refers to categories where no events were planted for that construct in that clone. Each field ID refers to a unique genetic construct (see Table 2).

Supplementary Table 3 Seed formation and seed viability for female clone 6K10. Yearly surveys checked for seed formation and seed viability from events which flowered.

Supplementary Table 4 Seed formation and seed viability for female clone 717. Yearly surveys checked for seed formation and seed viability from events which flowered.

Supplementary File 1 Sequences of gene fragments used to make RNAi constructs. A list of the portions of gene sequences used in creation of RNAi constructs.
