# Supplementary figures and images for: Phenotypic Expression and Stability in a Large-Scale Field Study of Genetically Engineered Poplars Containing Sexual Containment Transgenes
Source: Front Bioeng Biotechnol. 2018 Aug 3;6:100. doi: 10.3389/fbioe.2018.00100 (PMC6085431; doi:10.3389/fbioe.2018.00100)

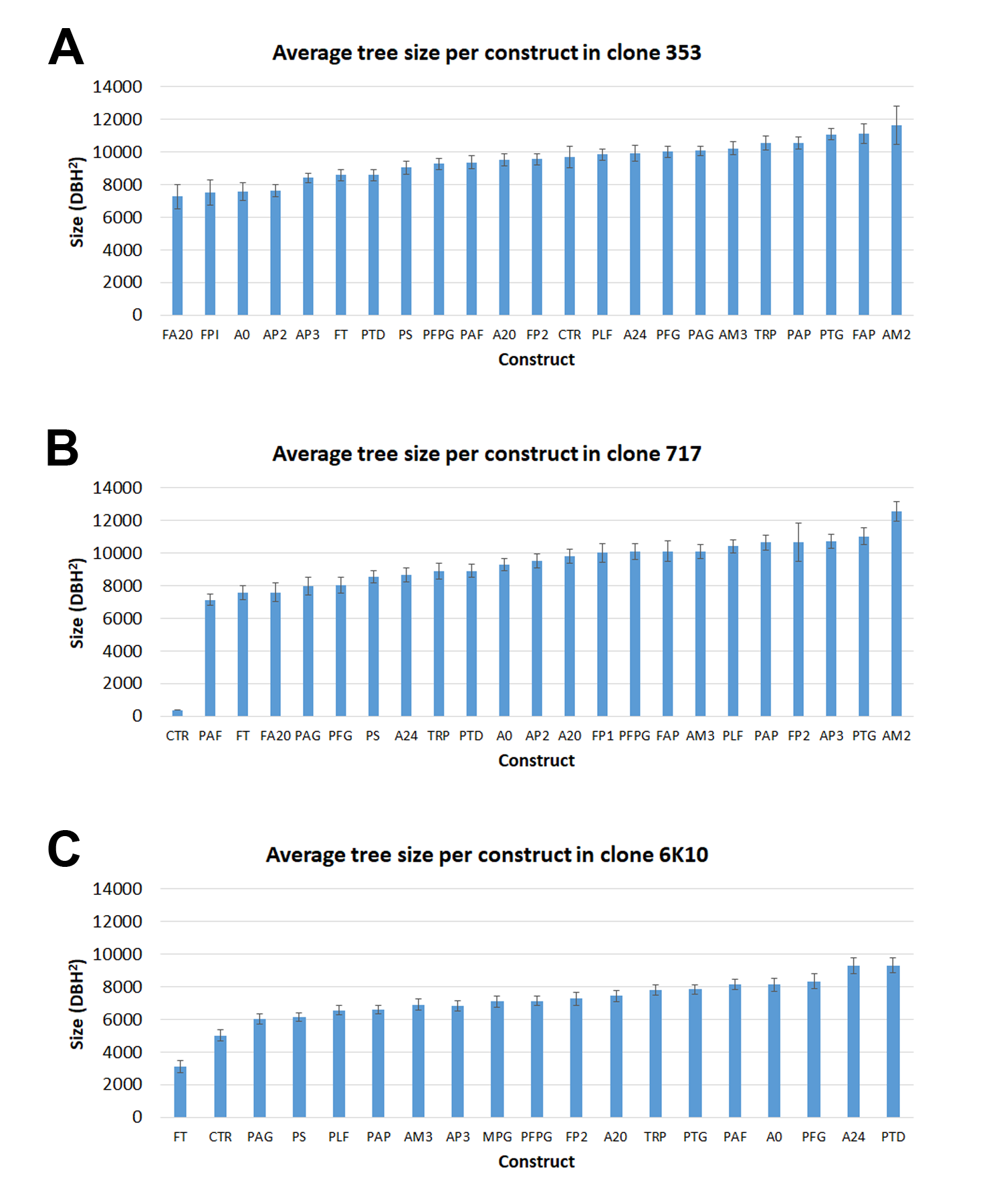

Supplement: Supplementary Figure 1 — Trees from most constructs performed well in all three clones. Average tree size (DBH2) of all trees was calculated from measurements collected in early 2018. Graphs show average size of all trees per construct for (A) clone 353, (B) clone 717 and (C) clone 6K10. Note that clone 717 had a single non-transgenic control tree (CTR) which grew poorly. Bars show construct averages across all trees; standard error of the mean is shown. [file Image_1.TIF]

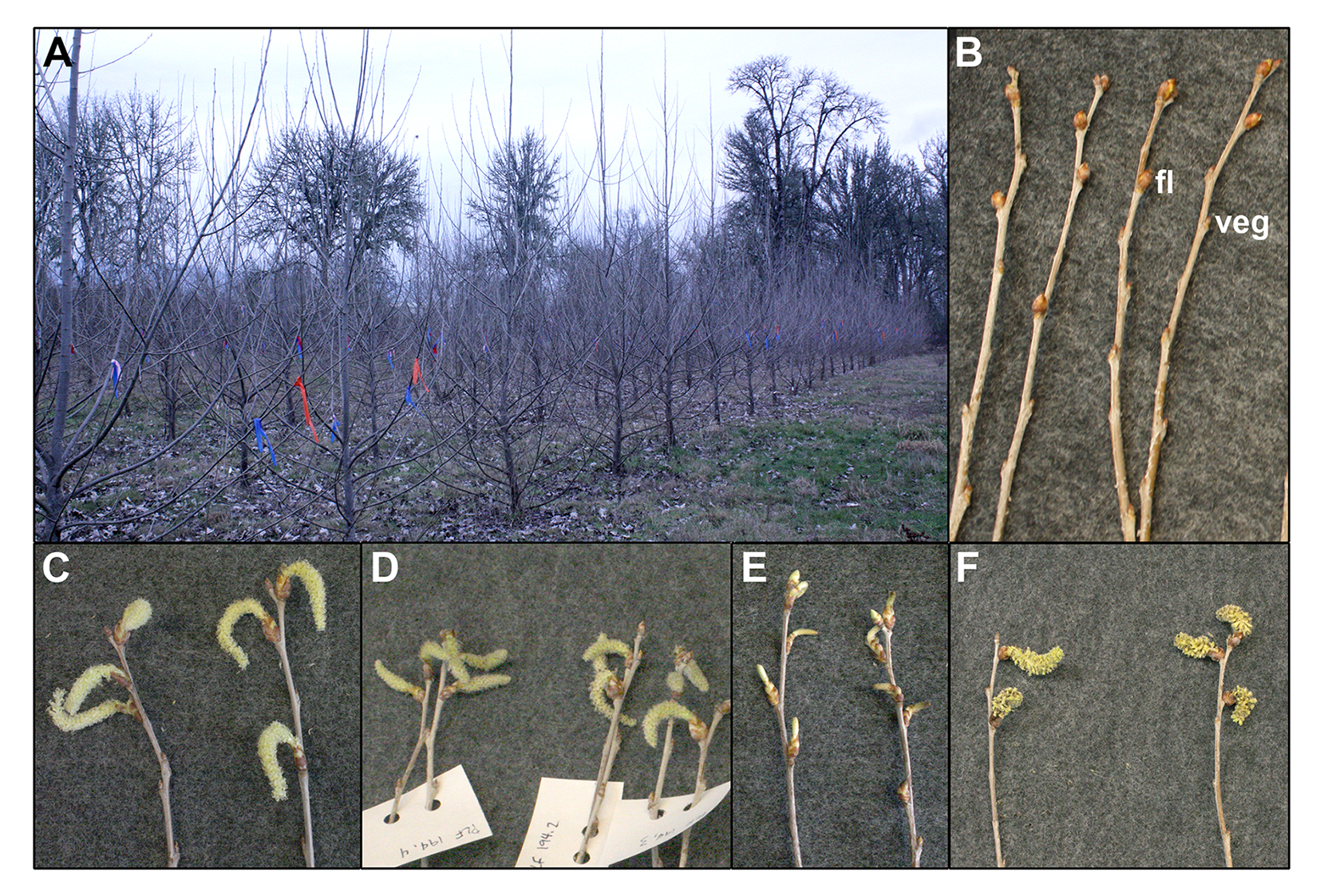

Supplement: Supplementary Figure 2 — Dormant floral buds were flushed in the lab for initial floral classification. (A) Trees from female clone 6K10 in January 2015, trees with floral buds have blue flagging, trees with buds collected for indoor analysis have an additional red flag. (B) Small twig cuttings with dormant floral (fl) and vegetative (veg) buds. (C) Flushed control catkins, (D) flushed normal RNAi-LFY catkins, (E) RNAi-LFY twigs with very small catkins, (F) RNAi-AG (mar) twigs with enlarged catkins. [file Image_2.TIF]
