# Supplementary material for: Phenotypic Expression and Stability in a Large-Scale Field Study of Genetically Engineered Poplars Containing Sexual Containment Transgenes
Source: Front Bioeng Biotechnol. 2018 Aug 3;6:100. doi: 10.3389/fbioe.2018.00100 (PMC6085431; doi:10.3389/fbioe.2018.00100)
Supplement: Supplementary Table 1 — Numbers of trees planted and survival to date by clone and construct. Trees were first planted in 2011 and survival monitored yearly. Current numbers of surviving trees are from the 2017 spring bud flush. Event refers to individual transgenic occurrences; ramets are individual trees, each field ID refers to a unique genetic construct (see Table 2). [file Table_1.PDF]

Field ID	Male clone 353			Female clone 717			Female Clone 6K10				
	Trees Planted	Trees Surviving	Events Planted	Trees Planted	Trees Surviving	Events Planted	Trees Planted	Trees Surviving	Events Planted	Total events	Total trees
AM2	15	10	4	48	47	14	0	0	0	**18**	**57**
AM3	70	61	19	66	64	19	60	58	15	**53**	**183**
AP2	43	41	12	60	58	17	0	0	0	**29**	**99**
AP3	72	71	20	67	65	20	59	59	15	**55**	**195**
CTR	18	9	0	2	1	1	24	24	0	**0**	**34**
A20	61	53	16	77	73	24	60	60	17	**57**	**186**
A0	22	17	10	58	55	15	54	54	13	**38**	**126**
A24	50	46	15	60	59	16	56	56	14	**45**	**161**
PTG	60	52	17	58	51	15	89	86	22	**54**	**189**
MPG	0	0	0	0	0	0	48	48	13	**13**	**48**
PAG	95	89	24	49	42	13	68	66	19	**56**	**197**
PAF	38	36	13	62	59	16	70	70	19	**48**	**165**
PAP	59	58	17	64	62	16	70	70	20	**53**	**190**
PTD	53	50	16	66	63	19	52	52	13	**48**	**165**
FAP	25	22	11	31	26	8	0	0	0	**19**	**48**
FA20	17	17	5	41	38	12	0	0	0	**17**	**55**
FT	66	60	16	63	62	16	24	23	6	**38**	**145**
PFG	58	51	14	60	56	16	40	40	10	**40**	**147**
TRP	36	34	9	48	42	12	90	86	25	**46**	**162**
PLF	59	57	18	75	72	22	60	59	15	**55**	**188**
PFPG	78	73	25	62	54	16	83	83	22	**63**	**210**
FPI	12	8	7	61	58	15	0	0	0	**22**	**66**
FP2	52	51	15	16	13	4	58	56	15	**34**	**120**
PS	53	46	13	60	60	15	74	73	20	**48**	**179**
Totals	1112	**1012**	316	1254	**1180**	341	1139	**1123**	295	**948**	**3315**
Survival to date			91.01%		94.10%				98.60%		**94.58%**

Supplementary Table 1: Numbers of trees planted and survival to date by clone and construct Trees were first planted in 2011 and survival monitored yearly. Current numbers of surviving trees are from the 2017 spring bud flush. Event refers to individual transgenic occurrences; ramets are individual trees, each field ID refers to a unique genetic construct (see Table 2).

	events planted			flowering events 2014			flowing events 2015			flowering events 2016			flowering events 2017			flowering events 2018		
construct	353	717	6K10	353	717	6K10	353	717	6K10	353	717	6K10	353	717	6K10	353	717	6K10
AM2	4	14	0	0	0	NA	0	11	NA	2	14	NA	3	13	NA	4	13	NA
AM3	19	19	15	0	1	14	2	13	13	11	16	15	13	17	14	17	18	15
AP2	12	17	0	0	1	NA	0	7	NA	6	14	NA	6	11	NA	8	15	NA
AP3	20	20	15	0	0	7	1	5	11	13	13	14	15	11	15	18	15	15
CTR	1	1	1	0	0	1	0	0	1	1	0	1	1	0	1	1	0	1
A20	16	24	17	0	0	9	2	15	12	14	22	17	13	21	17	15	24	17
A0	10	15	13	0	1	10	0	5	13	3	12	13	3	14	13	5	14	13
A24	15	16	14	0	0	7	2	11	10	14	15	14	14	14	14	15	15	14
PTG	17	15	22	0	2	21	3	11	21	12	15	22	14	15	22	16	15	22
MPG	0	0	13	NA	NA	9	NA	NA	12	NA	NA	12	NA	NA	12	NA	NA	12
PAG	24	13	19	0	0	10	6	11	15	23	12	19	21	12	19	23	13	19
PAF	13	16	19	0	1	13	4	12	18	9	15	18	11	14	18	9	16	18
PAP	17	16	20	0	0	12	2	15	18	14	16	19	14	16	20	16	16	19
PTD	16	19	13	0	1	12	7	16	13	15	17	13	16	15	13	15	17	13
FAP	11	8	0	0	0	NA	0	3	NA	7	8	NA	8	8	NA	6	8	NA
FA20	5	12	0	0	0	NA	0	6	NA	3	10	NA	3	10	NA	4	10	NA
FT	16	16	6	0	0	0	4	8	3	14	14	3	14	13	3	15	14	6
PFG	14	16	10	0	0	8	5	13	10	12	16	10	13	16	10	14	16	10
TRP	9	12	25	0	0	15	0	9	20	8	12	23	9	10	23	9	11	24
PLF	18	22	15	0	1	14	5	17	15	15	21	15	15	20	15	15	20	15
PFPG	25	16	22	0	0	19	5	11	20	22	16	22	23	15	22	23	16	22
FPI	7	15	0	0	0	NA	0	11	NA	2	15	NA	2	14	NA	2	15	NA
FP2	15	4	15	0	0	11	3	2	15	11	3	15	12	3	15	13	4	15
PS	13	15	20	0	1	5	2	3	7	4	7	12	6	4	18	13	7	20

Supplementary Table 2: Flowering events by clone, construct and year

Tree flowering was monitored yearly, events with at least one flowering tree were considered flowering. NA for flowering refers to categories where no events were planted for that construct in that clone. Each field ID refers to a unique genetic construct (see Table 2).

Data collected	Year				
	**2014**	**2015**	**2016**	**2017**	**total**
Total constructs	18	18	18	18	
Constructs flowered	17	18	18	18	
Constructs with seeds	7	16	15	12	
Seeds found	10	101	106	83	**300**
Seeds germinated	0	4	23	14	**41**

Supplementary Table 3: Seed formation and seed viability for female clone 6K10

Yearly surveys checked for seed formation and seed viability from events which flowered.

Data collected	Year				
	**2014**	**2015**	**2016**	**2017**	**total**
Total constructs	22	22	22	22	
Constructs flowered	8	22	22	22	
Constructs with seeds	2	20	7	6	
Seeds found	2	102	18	18	**140**
Seeds germinated	1	32	6	1	**40**

Supplementary Table 4: Seed formation and seed viability for female clone 717

Yearly surveys checked for seed formation and seed viability from events which flowered.
