# Supplementary material for: Phenotypic Expression and Stability in a Large-Scale Field Study of Genetically Engineered Poplars Containing Sexual Containment Transgenes
Source: Front Bioeng Biotechnol. 2018 Aug 3;6:100. doi: 10.3389/fbioe.2018.00100 (PMC6085431; doi:10.3389/fbioe.2018.00100)
Supplement: Supplementary File 1 — Sequences of gene fragments used to make RNAi constructs. A list of the portions of gene sequences used in creation of RNAi constructs. [file Data_Sheet_1.DOCX]

RNAi fragments:

>FT

GGTTATGGTGGACCCTGATGCACCCAGCCCAAGTGACCCCAGCCTAAGAGAATATTTGCATTGGTTGGTGACTGATATTCCAGCAACAACTGGGGCAAGCTTTGGCCATGAAACTGTGTGCTATGAGAGCCCGAGGCCGACAATGGGAATTCATCGGTTTGTTTTCGTCTTGTTTCGGCAACTGGGCAGGCAAACTGTGTATGCCCCTGGGTG

>PTAP1

AGATACTTGAACGCCACGAGAGGTATTCTTATGCAGAGAGGCAATTAGTCGCAACTGATCTTGATTCGCAGGGGAACTGGACCCTAGAGTATAACAGGCTCAAGGCAAAGGTTGAACTTCTACAGAGAAACCACAGGCACTATTTGGGGGAAGATCTGGACTCCGTGAGTCTCAAAGAGCTTCAAAACTTGGAGCAACAGATTGATACCGCCCTTAAACTCATTCGGGAAAGAAAAAACCATCTGATGTATCAGTCGATTTCTGAGCTGCAGATAAAGGAGAAGGCAATTAAAGAGCAAAATAACATGTTAGTGAAGCAGATCAAGGAGAAGGAGAA

>PTLF

TCATTGTAACAGAGCCTGGTGAAGTGGCACGTGGCAAAAAGAACGGTCTTGATTACCTCTTCCATTTATATGAACAGTGTCGTGATTTCTTGATCCAAGTCCAAAGCATTGCGAAGGAGAGGGGAGAAAAATGCCCCACTAAGGTGACAAATCAGGTGTTTAGGTATGCCAAGAAGGCAGGAGCAAGCTACATCAACAAGCCCAAAATGAGACACTACGTGCATTGCTATGCTTTACATTGCCTCGATGAGGACGCATCCAATGCACTTAGGAGAGCGTTCAAG

>AGL20/SOC1

CAGAGGCATGTGAAAGAAAGCAACACAAACAAGCAGACAAGCGAACTGAACATGGAGCAACTGAAGGGCGAAGCAGCTAGCATGATAAAGAAGATAGAGATTCTTGAAGTTTCGAAACGAAAGCTACTGGGAGAATGTTTGGGATCGTGCACTGTTGAAGAACTGCAACAAATCGAACAACAGTTAGAGAGGAGTGTAAGCACCATCCGAGCTAGAAAGAATCAGGTTTTCAGAGAACAGATTGAGCAACTAAAACAAAAGGAGAAGCAACTGACAGCTGAAAATGCAAGGCTGTCTAACAAGTCTGGCGTCCAGCCATGGCGAGTACTCTCAAGGGAACAGAGAGAGAATTTACCCTGTGAAGAACAGAGAGATAGCAGTTCAATTTCGGATGTGGAGACCGAATTGTTC

>AGL24

CCGCACTCCAATAACCTCGACAAAATAAATCCGCCGTCTCTTGAGTTGCAGCTAGAAAACAGCAATCACATGCGATTGAGCAAGGAAGTTTCCGAGAAGAGTCATCAGCTAAGGCGGATGAGAGGTGAAGATCTTCACGGACTAAATATAGAAGAATTGCAGCAATTGGAAAAGGCGCTTGAAGTAGGACTTAGCCGCGTGCTTGAAACCAAGGGAGAACGAATTATGAATGAGATATCTACCCTTGAAAGGAAGGGAGTACAGCTTTTGGAAGAGAATAAGCAACTAAAACAGAAGATCGCAACCATTTACAAGGGAAAAGGACCCGCCCTTGTCGATTTAGACACAGCAGTTCAGGAAGAAGGGATGTCATCGGAGTCTACAACCAATGTTTGCAGCTGCAGCAGTGGCCCTCCTGTGGAGGATGATAGCTCCGA

>PTAG

GGGTCAGTTTCTGAAGCCAATGCTCAGTTCTATCAGCAAGAAGCTGCCAAGCTGCGCTCGCAAATTGGTAATTTGCAGAATTCAAACAGGAATATGCTGGGTGAATCACTTAGTGCATTGAGTGTGAAGGAACTTAAGAGCTTGGAGATAAAACTTGAGAAAGGAATTGGTAGAATTCGTTCGAAAAAGAATGAGCTGTTGTTTGCTGAAATTGAGTATATGCAGAAGAGGGAGATTGACTTGCACAACAATAACCAGCTTCTCCGAGCAAAGATTGCAGAGAATGAAAGAAAGCGACAGCACATGAATTTGATGCCGGGAGGTGTCAACTTCGAGATCATGCAGTCTCAACCATTTGACTCTCGGAACTATTCTCAAGTTAATGG

>PTD

TATCAGAACGCTTTAGGCATAGATCTGTGGGGCACTCAATACGAGAAAATGCAAGAGCACTTGAGGAAGCTGAATGATATCAATCATAAGCTGAGACAAGAAATCAGGCAGAGGAGAGGAGAGGGCCTGAATGATCTGAGCATTGATCATCTGCGCGGTCTTGAGCAACATATGACTGAAGCCTTGAATGGTGTGCGTGGCAGGAAGTACCATGTGATCAAAACACAAAACGAAACCTACAGGAAGAAGGTGAAGAATTTAGAGGAGAGACATGGAAACCTCTTGATGGAATATGAAGCAA

>FPFL1

GGAGAAAGGTATTGGTGCACTTGCCATCAGGCCAAGTGGTATCCTCATACTCTTCCCTTGAACAGATCTTGAACGAGTTAGGATGGGAGAGGTACTATGGAGGTGACCCTGACCTCTTCCAATTCCACAAGCAATCTTCCATTGA

>FPFL2

GGAGAACCCAGGAGCTGAATCACTAGATGGAAGCCGGCAAGGGTCAAGTATGCGGCGAAAAGTGCTAGTTCACTCCCCTAGTAATGAGGTTATAACTTCTTATGCCGTTCTTGAACGCAAGCTATATTCTCTTGGGTGGGAGAGGTACTATGATGATCCGGATCTCCTTCAATTCCACAAAAGATCAACTGTTCATCTCATCTCTCTCCCTAAGGATTTTAACAAGCTCAGGTCCATGCA
